# Insulin-like growth factor-1 prevents miR-122 production in neighbouring cells to curtail its intercellular transfer to ensure proliferation of human hepatoma cells

**DOI:** 10.1093/nar/gku346

**Published:** 2014-05-09

**Authors:** Sudarshana Basu, Suvendra N. Bhattacharyya

**Affiliations:** RNA Biology Research Laboratory, Molecular and Human Genetics Division, CSIR-Indian Institute of Chemical Biology, Kolkata 700032, India

## Abstract

miRNAs are 20–22 nt long post-transcriptional regulators in metazoan cells that repress protein expression from their target mRNAs. These tiny regulatory RNAs follow tissue and cell-type specific expression pattern, aberrations of which are associated with various diseases. miR-122 is a liver-specific anti-proliferative miRNA that, we found, can be transferred via exosomes between human hepatoma cells, Huh7 and HepG2, grown in co-culture. Exosomal miR-122, expressed and released by Huh7 cells and taken by miR-122 deficient HepG2 cells, was found to be effective in repression of target mRNAs and to reduce growth and proliferation of recipient HepG2 cells. Interestingly, in a reciprocal process, HepG2 secretes Insulin-like Growth Factor 1 (IGF1) that decreases miR-122 expression in Huh7 cells. Our observations suggest existence of a reciprocal interaction between two different hepatic cells with distinct miR-122 expression profiles. This interaction is mediated via intercellular exosome-mediated miR-122 transfer and countered by a reciprocal IGF1-dependent anti-miR-122 signal. According to our data, human hepatoma cells use IGF1 to prevent intercellular exosomal transfer of miR-122 to ensure its own proliferation by preventing expression of growth retarding miR-122 in neighbouring cells.

## INTRODUCTION

miRNAs are ∼22 nucleotide long non-coding RNA molecules which act as key post-transcriptional regulators of gene expression in metazoan animals and plants. miRNAs repress gene expression by binding to complementary sequences in the 3’untranslated region (UTR) of target mRNAs, thereby inhibiting translation and inducing deadenylation and degradation of target mRNAs (1). miRNA biogenesis is regulated both at transcriptional and post-transcriptional level and misregulation of these processes leads to various human pathologies, including cancer (2). Expression profiles of miRNAs revealed a cancer-type specific signature of miRNA expression that differ with disease progression stages (3–6). Among the miRNAs expressed in animal cells, some miRNAs can act as tumour suppressors while increased expression of few other miRNAs can cause transformation of cells and cancer in mouse models (7,8).

In a tumour microenvironment, cancer cells interact with normal non-transformed cells and compete for resources and factors in their environment. Interestingly, non-tranformed cells may have an inhibitory role against the growth and proliferation of transformed tumour cells. Previously, it was demonstrated that Normal breast epithelial cells and their Conditioned Media (CM) could inhibit proliferation of a variety of breast cancer cell lines (9).

Recently, it has been shown that miR-143, a tumour suppressor miRNA, released from normal prostrate cells can transfer growth inhibitory signals to prostrate cancer cells (10). Thus the normal cells secrete anti-proliferative miRNAs in an attempt to maintain normal miRNA homeostasis; however the abnormal cancer cells finally circumvent this inhibitory effect resulting in expansion of the tumour.

miRNAs have been detected in various human body fluids including peripheral blood plasma, saliva, serum and milk (11). Tumour associated miRNAs were higher in serum of lymphoma patients as compared to healthy controls (12) while miRNA content of mast cell derived exosomes are transferable to other human and mouse mast cells (13). Epstein-Barr virus (EBV) infected B cells secrete EBV encoded miRNAs in exosomes which repress immunoregulatory genes (14). Exosomal miRNAs are released through a ceramide-dependent secretory machinery and the secreted miRNAs are transferable and functional in the recipient cells (15). In a recent study, exosome mediated delivery of oncogenic miRNAs and regulation of invasiveness of breast cancer cells by macrophages has been reported (16). THP-1-derived microvesicles that can enter and deliver miR-150 into human HMEC-1 cells reduced c-Myc expression and enhanced cell migration of HMEC-1 cells (17). Exosomal miRNA transfer from T cells to Antigen Presenting Cells in immune synapses was also documented (18). These and other reports indicate that cells communicate with each other by secreting miRNA laden vesicles that serve as intercellular messengers.

miR-122 has been characterized for its multiple roles in liver physiology, metabolism and in modulation of hepatitis C virus replication. It is a liver-specific miRNA representing 70% of the liver miRNA population (19,20). Notably, its loss or downregulation has been associated with human and rodent hetatocellular carcinoma (HCC) development and progression (21–27). In this study, we have documented exosome mediated transfer of miR-122 between co-cultured human hepatoma cells. HepG2 and Huh7 are two human hepatic cell lines that are well explored to study liver function and metabolism. HepG2 cells have highly reduced levels of miR-122 whereas Huh7 cells express this hepatic miRNA in high amounts (28,29). miR-122 transfer from Huh7 to HepG2 can change the expression of various miR-122 regulated genes in the recipient HepG2. There is a concomitant downregulation of miR-122 expression in Huh7 cells mediated by HepG2 secreted Insulin-like Growth Factor 1 (IGF1). HepG2 cells overcome the restorative effect exerted by the transferred miR-122 by secreting IGF1 which in turn inhibits miR-122 biogenesis in neighbouring cells. This reciprocal effect exerted by HepG2 on miR-122 producing neighbouring cells may a indicate a strategy that hepatic cancer cells adopt to modulate their microenvironment to their benefit and proliferation.

## MATERIALS AND METHODS

### Cell culture

Human HCC cell lines HepG2 and Huh7 were cultured in Dulbecco's modified Eagle's medium (DMEM; Gibco-BRL) supplemented with 10% fetal bovine serum (FBS; GIBCO-BRL) and Penicillin Streptomycin (1X) antibiotics (GIBCO).

### Plasmid constructs

The RL reporters (Renilla luciferase) were previously described (30,31). Details of plasmids are available in Supplementary Table S1.

siControl, siSMNaseII, siIGF1R and siIGF1 were used for transfection at 100 pmoles per well of a confluent six-well plate. miR-122 and let-7a mimic and anti-let-7a and anti-miR-122 were purchased from Ambion and was used at 100 pmoles to transfect cells per well of a six-well plate. Cells were differentially transfected for fluorescence-activated cellsorting (FACS) and microscopy using pcIneoGFP and DsRed Monomer (Clontech) plasmids. To exogenously express miR-122, cells were transfected with pmiR122 plasmid. For transfections, 1 μg of each of the plasmids was used for transfection of 10^6^ cells in a 10 cm^2^ well. Details of plasmid and siRNA constructs are given as Supplementary Tables S1 and S2.

### Luciferase assays

For luciferase assays 10^6^ cells in a 10 cm^2^ well were transfected with either RL-con or RL-per-miR-122 plasmids (above) in parallel sets. To detect fold repression in HepG2, 10^6^ cells in a 10 cm^2^ well were transfected with 150 ng of each of the plasmids. Normalisation was done with a Firefly (FF) luciferase construct which was co-transfected along with the RL constructs (1 μg for 1 × 10^6^ cells). After 24 h of transfection, cells were co-cultured with Huh7 and HepG2 cells in a 24 well-plate at desired Huh7:HepG2 ratios. These were then co-cultured for 48 h. After that cells were lysed with 1 X Passive Lysis Buffer (Promega). Renilla (RL) and Firefly (FL) activities were measured using a Dual-Luciferase Assay Kit (Promega) following the suppliers protocol on a VICTOR X3 Plate Reader with injectors (Perkin Elmer). Mean Fold Repression was calculated by dividing the FF normalized RL-Con value with that of FF-normalized RL-per-miR-122 value. Relative fold repression was calculated by taking the control mean fold repression as 1. For co-culture-based luciferase assays in HepG2, cells were usually co-cultured at a ratio of 40% of HepG2 to 60% of the co-cultured cell.

To detect fold repression in Huh7, 10^6^ Huh7 cells in a 10 cm^2^ well were transfected with 100 ng of RL-Con and RL-perf-miR-122. For normalisation of RL expression, cells were co-transfected with 500 ng of the FF luciferase construct and after 24 h of transfection, cells were splited and co-cultured with other nontransfected cells at a ratio of 50% of transfected Huh7 and 50% of the nontransfected cell. After 72 h, cells were lysed with 1 X Passive Lysis Buffer and luciferase levels were detected in a Victor X3 plate reader. All luciferase assays used in this study have been done in triplicate. All experiments were performed minimum three times before the SD values were calculated.

### Sorting of co-cultured and artificially mixed HepG2 and Huh7 populations

For FACS analysis, co-cultured HepG2 and Huh7 cells were used. HepG2 cells (5 × 10^5^) were added to equal number of Huh7 cells in a 20 cm^2^ dish and co-cultured for 48 h. As a control 5 × 10^5^ HepG2 cells and 5 × 10^5^ Huh7 cells were seeded separately in 10 cm^2^ dishes. Seeding was done such that cells attained 100% confluencies after 48 h. After 48 h, co-cultured cells were trypsinized and collected for FACS analysis. HepG2 and Huh7 cells seeded separately were also similarly collected and mixed together to comprise the artificially mixed cell population which was used as a control to negate any false positive signals during the sorting process. For sorting, co-cultured and artificially mixed cells were collected as described above. Cells were resuspended in FACS-buffer (PBS with 3% FBS) to 2 × 10^6^ cells/ml. Cells were then subjected to sorting in a BD FACS Aria II sorter and sorted cells were collected in FBS coated FACS tubes containing FACS buffer.

To measure cell growth rate, co-cultured and artificially mixed cells were collected by trypsinization at specific time points. Cells were then fixed with 4% paraformaldehyde for 30 min. Fixed cells were kept in 75% ethanol. Just before the sorting experiment, cells were rehydrated in 1 X PBS for 30 min. In a population the percentage of Green Fluorescent Protein (GFP) and DsRed positive cells were estimated by FACS analysis. Relative growth rate was measured by dividing the percentage of green (GFP)/Red (DsRed) cells in the co-cultured set with the percentage in the control set.

### Cell transfections

All transfections (plasmids, siRNAs and 2’-O-methyl oligos) were performed using Lipofectamine 2000 (Invitrogen) following manufacturer's instructions.

### Quantitative estimation of mRNA and miRNA levels

RNA was extracted by using the TRIzol reagent according to the manufacturer's protocol (Invitrogen). Real-time analyses by two-step RT-qPCR was performed for quantification of miRNA and mRNA levels. All RT-qPCRs were performed on a 7500 REAL TIME PCR SYSTEM (Applied Biosystems). mRNA real-time quantification was generally performed in a two-step format using Eurogentec Reverse Transcriptase Core Kit and MESA GREEN qPCR Master Mix Plus for SYBR Assay with Low Rox kit from Eurogentec following the suppliers’ protocols. The comparative C_t_ method which typically included normalisation by 18S rRNA levels for each sample was used for relative quantification. Details of mRNA gene specific primers are given as Supplementary Table S3.

Quantification of miRNA levels was done using Applied Biosystem TaqMan® chemistry based miRNA assay system. Assays were performed with 25 ng of cellular RNA and 100 ng of exosomal RNA unless specified otherwise, using specific primers for human miR-122, miR-24 and let-7a (assay ID 000445, 002440, 000377, respectively). U6 snRNA (assay ID 001973) was used as an endogenous control. One-third of the reverse transcription mix was subjected to PCR amplification with TaqMan® Universal PCR Master Mix No AmpErase (Applied Biosystems) and the respective TaqMan® reagents for target miRNA. The RT reaction condition was 16°C, 30 min; 42°C, 30 min; 85°C, 5 min; 4°C, ∝. The PCR condition was 95°C, 5 min; 95°C, 15 s; 60°C, 1 min; for 40 cycles.

Samples were analysed in triplicates from minimum two biological replicates. The concentrations of intra and extracellular miRNAs were calculated based on their normalized *C_t_* values. The ΔΔCt method for relative quantitation (RQ) of gene expression was used and relative quantification was done using the equation 2^−ΔΔCt^ (as per ‘Guide to Performing Relative Quantitation of Gene Expression Using Real-Time Quantitative PCR’ obtained from the Applied Biosystems website, http://www3.appliedbiosystems.com/cms/groups/mcb_support/documents/generaldocuments/cms_042380.pdf).

### Exosome isolation and experiments with CM

For all exosome-related experiments, exosome depleted FCS was used. Exosome depleted FCS was either commercially obtained (System Biosciences Catalog no. EXO-FBS-250A-1) or prepared by ultracentrifugation of the FCS used at 110,000×g for 5 h. For exosome isolation cells were grown in media made from exosome depleted FCS. The supernatant CM from two 60 cm^2^ plates, having 6 × 10^6^ donor cells (Huh7 or HepG2) each were taken. The CMs were centrifuged first at 300×g for 10 min, then at 2000×g for 15 min followed by centrifugation at 10,000×g for 30 min. All centrifugations were done at 4°C. The CM was then filtered through a 0.22 μm filter unit. This was then centrifuged at 100,000×g for 90 min at 4°C. After centrifugation, the supernatant was discarded. The pellet was resuspended in media and added back to recipient cells (HepG2 or Huh7) in a 24-well plate format such that 2 × 10^5^ recipient cells received the exosomes from 1 × 10^6^ donor cells. For CM-based assays the same ratio was followed with CM from 10^6^ cells being added to 2 × 10^5^ cells. For incubation times greater than 24 h, media was replaced with fresh CM after every 24 h. For the isolation of miR-122, anti-miR-122 and anti-let7a carrying exosomes, 1 × 10^6^ cells were transfected and 24 h after transfection, the cells were reseeded onto a 60 cm^2^ plate. Cells were grown for 48–72 h and exosomes isolated from the CM of these cells.

**Figure 1.**
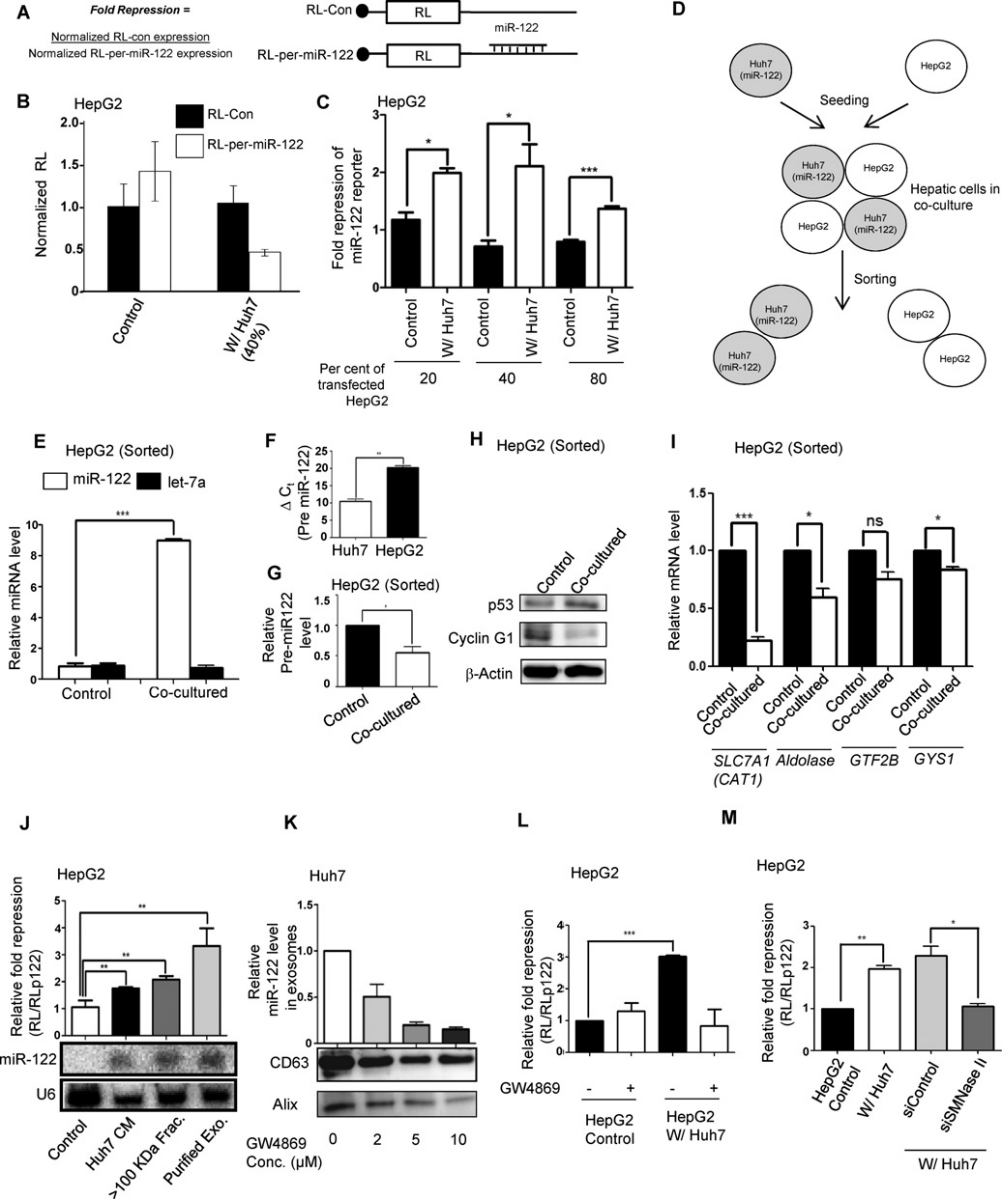
Huh7 cells can transfer miR-122 to neighbouring HepG2 cells in co-culture. (**A**) Schemes of RL reporters used for miR-122 activity analysis in hepatic cells. (**B, C**) Effects of variable cell-to-cell ratios of Huh7 and HepG2 in co-culture on miR-122 activity transfer to HepG2 cells. Normalized RL values for individual reporter transfected HepG2 cells co-cultured either with 40% of non-transfected HepG2 (control) or Huh7 were plotted (B). Mean fold repression was estimated by dividing the normalized RL levels in RL-con and RL-per-miR-122 expressing cells with changing Huh7 to HepG2 cell number ratios. Experiments were done in triplicate (C). Data shown are the mean ±SEM. Relative fold repression was determined by setting the repression level of control as 1. (**D**) Flowchart of co-culture followed by sorting experiment. GFP positive HepG2 cells were co-cultured with DsRed and miR-122 expressing Huh7 cells and after 48 h, cells were FACS sorted and were used for further analysis. (**E**) Let-7a and miR-122 levels in sorted HepG2 cells obtained as described in D. Relative miRNA levels were measured by quantitative RT-PCR. Normalization was done by U6 snRNA. HepG2 cells, grown separately but pre-mixed with Huh7 immediately before the sorting, were used as control. Data shown are the mean ±SEM from three separate experiments performed in triplicate. (**F**) Relative level of pre-miR-122 in Huh7 and HepG2 cells. Same amount of RNA isolated from these cells was used for analysis. 18S rRNA was taken as the internal control and ΔCt ( = C_t sample_- C_t 18S_) values were plotted. (**G**) Relative levels of pre-miR-122 were detected in Sorted HepG2 cells obtained as described in D*.* Normalization of qPCR data was done by 18S rRNA. Data shown are the mean ±SEM from three separate experiments performed in triplicate (lower panel). (**H**) Cyclin G1 and p53 expression in sorted HepG2 cells both in control or co-cultured with Huh7 for 48 h. β-actin was used as loading control. (**I**) Relative expression of miR-122 target genes in sorted HepG2 cells measured by real-time quantitative PCR. Normalization of qPCR data was done by 18S rRNA. Data shown are the mean ±SEM from three separate experiments performed in triplicate. (**J**) Repression of miR-122 reporter in HepG2 cells treated either with Huh7 CM, or >100 KDa cutoff fraction of Huh7 CM, or with exosomes isolated from Huh7 cells (top). miR-122 levels are detected in the bottom panel. U6 serves as loading control. (**K**) Immunoblotting of Alix and CD63 and quantification of miR-122 in exosomes secreted by Huh7 cells treated with increasing amounts of the neutral Sphingomyelinase II inhibitor GW4869. (**L**) Levels of miR-122-mediated repression in reporter transfected and Huh7 co-cultured HepG2 cells in presence and absence of GW4869. (**M**) miR-122-mediated repression in HepG2 cells co-cultured with Huh7 cells transfected with a control siRNA or siRNA against neutral Sphingomyelinase II. Data are presented as means ±SEM in all results obtained from multiple experiments (*n* = 3) when ns: non-significant, *P < 0.05, **P < 0.01 and ***P < 0.001. All luciferase experiments have been conducted in triplicate. For data presented in panels E, F, G, I and K the ΔΔCt method for RQ of gene expression was used and generated using the equation 2^−ΔΔCt^. *P* values were determined by paired t test.

For the experiment in Figure 1J, the supernatant from Huh7 cells was centrifuged to clear cellular debris and this constituted the Huh7 CM. This was then further centrifuged in a centricon with a molecular weight cutoff of 100 kDa such that constituents having molecular weight of <100 kDa were filtered out and those which were >100 kDa were retained. The latter was then filter sterilized and added to HepG2 cells.

For the experiment described in Figure 1K, CM from two 60 cm^2^ plates, after being passed through the 0.22 μm filter was loaded on to a 30% sucrose cushion. This was ultracentrifuged at 100 000xg for 90 min at 4°C. The medium above the sucrose cushion was discarded leaving behind a narrow layer of medium with the exosomes at the interface. 1XPBS was added and the separated exosomes were washed at 4°C for 90 min at 100 000xg. The pellet was resuspended in 200 μl of 1X Passive Lysis Buffer, half of which was used for western analysis and the remaining half was used for miR-122 analysis by real-time quantification.

### Incubation with growth factors

Various growth factors were purchased from Invitrogen. They are Recombinant Human IGF-1 (Cat no. PHG0078), Recombinant Human TGF-β1 (Cat no. PHG9214), Recombinant Human HGF (Cat No. PHG0254), Recombinant Human IGF-II (Cat no. PHG0084), Recombinant Human EGF (Cat no. PHG0315).

For Figure 5B, cells transfected with RL reporters were incubated overnight with media (DMEM) containing 0.1% FCS. This was replaced the next day with DMEM containing indicated concentrations of various growth factors. Cells were lysed after 24 h. For all other experiments with growth factors, indicated concentrations of the factors were added to Huh7 CM which had been depleted for miR-122 containing exosomes by centrifuging at 100,000 x g for 90 min. Incubations were done for 72 h with addition of fresh media (exosome depleted) with IGF1 once in between. For antibody blocking experiment described in Figure 5H, indicated concentrations of antibodies were added for 1 h at 37°C to HepG2 CM. Following this pre-incubation, the antigen–antibody mixture was added to reporter transfected Huh7 cells.

**Figure 2.**
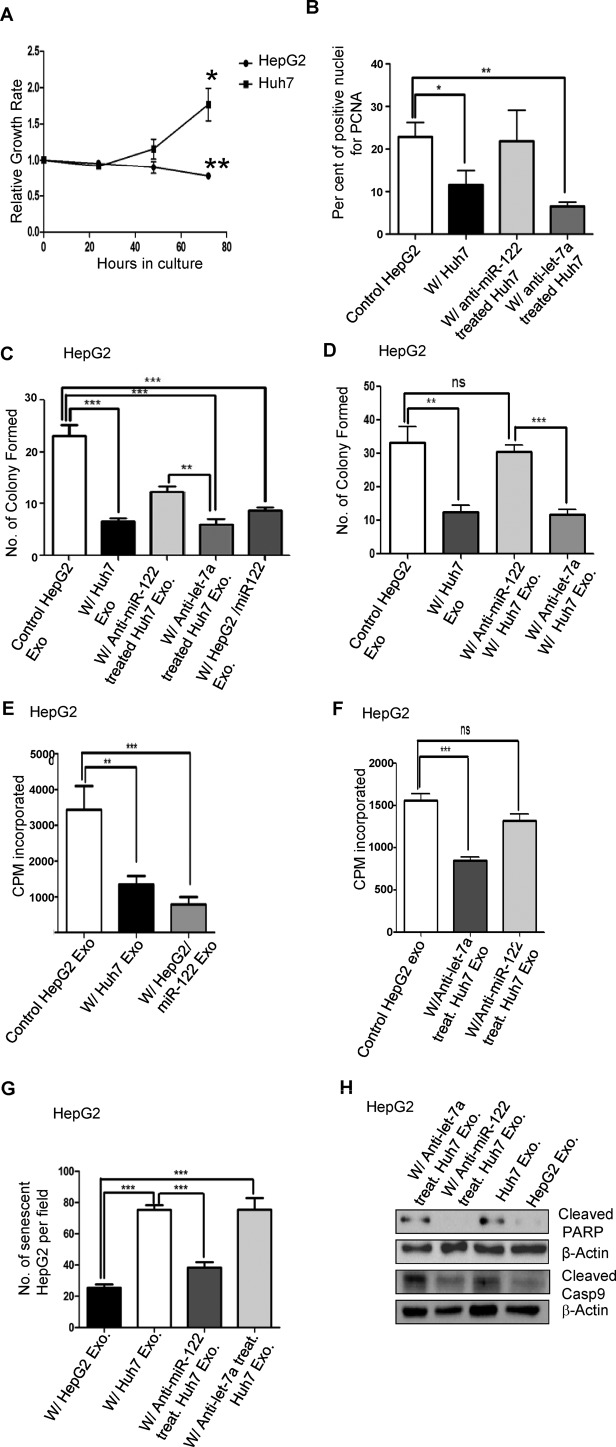
HepG2 cells receiving miR-122 from the donor Huh7 cells exhibit a decreased growth rate, increased senescence and increased sensitivity to apoptosis inducing agent. (**A**) HepG2 cells expressing GFP were co-cultured with equal number of Huh7 cells expressing DsRed. As a control, equal numbers of GFP-HepG2 and DsRed-Huh7 cells were cultured separately for same durations of time. Cells were collected by trypsinization and fixed and analysed by FACS. The percentage of GFP positive and DsRed positive cells in each sample were scored and relative growth rates were plotted. Relative growth rates were calculated by dividing the number of green (GFP) or Red (DsRed) cells in co-cultured sets by their number in the corresponding control sets. *P* values were determined by paired t test between 0 and 72 h replicates. *n* = 4. (**B**) Number of PCNA positive DsRed expressing HepG2 cells in co-culture either with HepG2 cells (control, not expressing DsRed) or with Huh7 cells (untreated or expressing anti-miR-122 or anti-let-7a oligos). PCNA (mitosin) positive cells were then detected by indirect immunofluorescence. Data shown represents three independent slides with five fields from each slide. *P* values were calculated using unpaired t test. (**C**) HepG2 cells treated with miR-122 containing Huh7 exosomes showed reduced growth and size of colonies. HepG2 cells were incubated with exosomes from HepG2, Huh7, anti-miR-122 or anti-let-7a transfected Huh7, and HepG2 cells expressing miR-122, for 7 days with changes after every 48 h. The cells were then reseeded at 1 × 10^3^ cells/cm^2^. After 96 h the numbers of colonies formed in each case were counted. For each set *n* = 3. *P* values were calculated using unpaired t test. (**D**) Experiments were redone with HepG2 cells transfected with anti-miR-122 and anti-let7 oligonucleotides. For each set *n* = 3. *P* values were calculated using unpaired t test. (**E**) Tritiated thymidine incorporation in HepG2 cells, incubated with exosomes from control HepG2, Huh7 and HepG2 expressing miR-122 and grown in presence of ^3^H labelled thymidine. Experiment was done in three sets. *P* value was calculated by using unpaired t test. (**F**) Similar experiments were done with Huh7 exosomes isolated from anti-miRNA transfected Huh7 cells. For each condition *n* = 3. *P* value was calculated by using unpaired t test. (**G**) Estimation of number of senescent HepG2 cells per field. Cells were incubated with exosomes from control HepG2, Huh7, Huh7 transfected with anti-miR-122 or anti-let-7a oligos for 24 h. Cells were then fixed and senescence assays were performed. Data represents three independent slides with five fields from each slide. *P* value is calculated using unpaired t test. (**H**) Western analysis of cleaved PARP and Caspase 9 in HepG2 cells treated with 50 μg/ml doxorubicin along with exosomes isolated from HepG2 (Control), Huh7, or anti-miR-122 and anti-let7 transfected Huh7. Exosome treatment was done for 48 h with one change after 24 h when Doxorubicin was added. Data are presented as mean ±SEM in all results obtained from multiple experiments (minimum three) when ns: non-significant, *P < 0.05, **P < 0.01 and ***P < 0.001.

**Figure 3.**
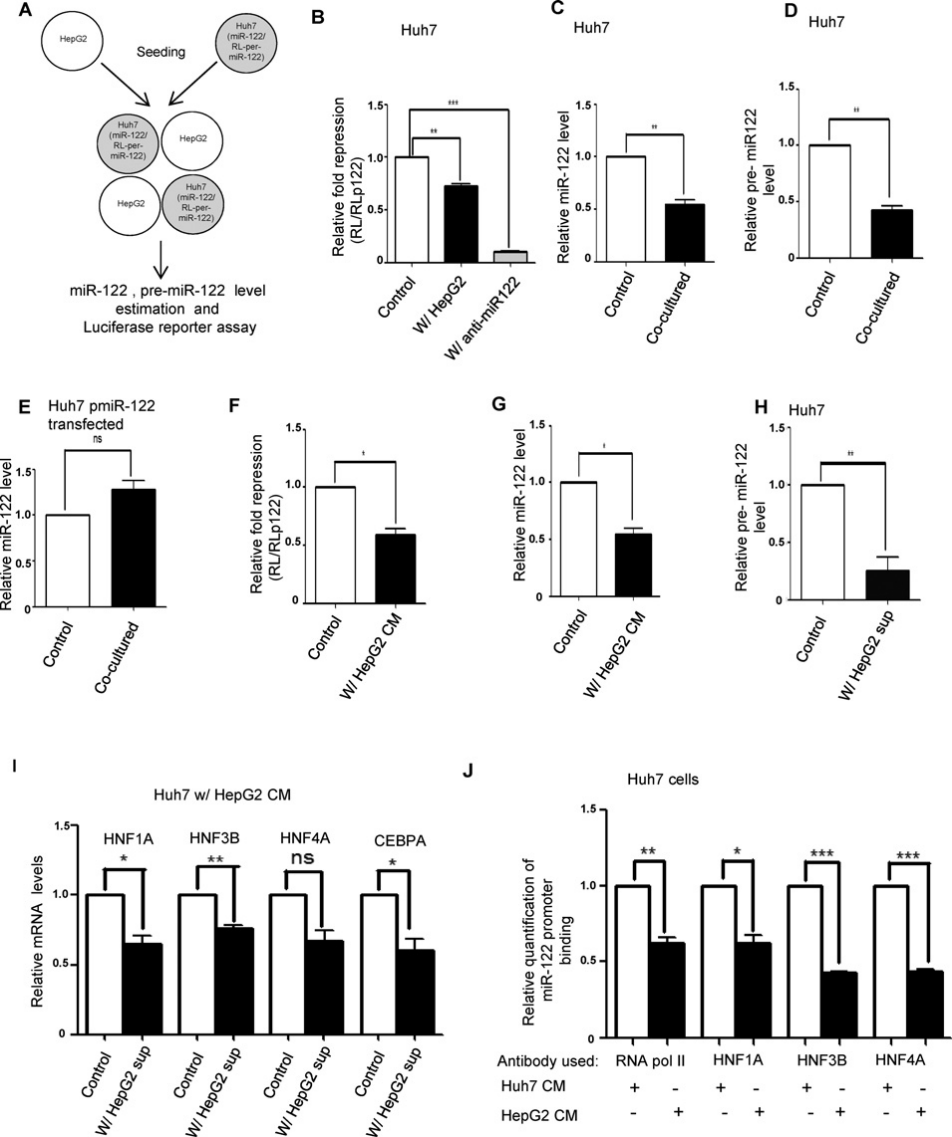
HepG2 cells secrete factors to reduce expression of miR-122 in hepatic cells. (**A**) Schemes of co-culture of HepG2 and Huh7 cells expressing RL reporter for miR-122. (**B**) Effect of co-culture on miR-122 activity in Huh7 cells expressing RL reporter. Huh7 cells were co-cultured with non-transfected Huh7 cells (as control) or HepG2 cells and after 72 h of co-culture, cells were lysed and luciferase activity was measured. Fold repression was estimated by dividing the normalized RL levels in RL-con and RL-per-miR-122 expressing cells. Relative fold repression was determined by setting the repression level of control as 1. Experiments were performed in triplicate and *P* value was calculated by using paired t test. (**C**) Levels of miR-122 in Huh7 cells grown separately or co-cultured with HepG2 cells at ratios of 1:1. For control, equal number of HepG2 and Huh7 cells were cultured separately for the same duration and were mixed together just before lysis. RNA was isolated from the control and co-cultured sets and real-time qPCR was performed to detect miR-122 level change. Data represents three independent experiments with qPCR for each experiment being conducted in triplicate. *P* values were calculated by paired t test. (**D**) RNAs obtained in experiments described in panel C were subjected to real-time quantification to estimate the relative pre-miR122 level in the control and co-cultured samples. Data represents three independent experiments with qPCR for each experiment being conducted in triplicate. *P* values were calculated by paired t test. (**E**) Real-time qPCR analysis was done to detect the level of miR-122 in pmiR-122 plasmid transfected Huh7 in control and HepG2 co-cultured Huh7 cells. Huh7 cells were transfected with miR-122 expressing pmiR-122 plasmid that drives pre-miR-122 expression from a U6 promoter. Data represents four independent experiments with qPCR for each experiment being conducted in triplicate. *P* values were calculated by paired t test. (**F**) miR-122-mediated repression in Huh7 cells transfected with RL reporter and incubated with either Huh7 (control) or HepG2 CM. Experiments were performed in triplicate and *P* value was calculated by using paired t test. (**G**) Real-time qPCR analysis to detect miR-122 level change in Huh7 cells treated with HepG2 CM for 72 h. As control, Huh7 cells were treated with Huh7 CM. Data represents three independent experiments with qPCR for each experiment being conducted in triplicate. *P* values were calculated by paired t test. (**H**) Real-time analysis of pre-miR122 level in Huh7 CM (control) and HepG2 CM treated Huh7 cells. Data represents six independent experiments with qPCR for each experiment being conducted in triplicate. *P* values were calculated by paired t test. (**I**) QRT-PCR-based quantification of expression level changes of various hepatic nuclear factors in Huh7 cells treated with CMs from Huh7 (control) or HepG2 cells. Data represents four independent experiments with qPCR for each experiment being conducted in triplicate. *P* values were calculated by paired t test. (**J**) Chromatin immunoprecipitation assays followed by quantitative real-time PCR to detect the *in vivo* interaction between three HNFs (HNF1α, HNF3β and HNF4α) and the miR-122 promoter in Huh7 cells incubated with either Huh7 CM (Control) or HepG2 CM. Huh7 cell chromatin fragments were immunoprecipitated with antibodies for each HNF and RNA pol II. Data represents three experimental sets with qPCR for each set being done in triplicate. Relative quantification of miR-122 promoter binding by HNFs was done by the formula 2^−ΔCt^ where ΔC_t_ was calculated by subtracting the C_t_ for each HNF associated DNA in Huh7 CM treated set from the corresponding HepG2 CM treated set. *P* values were determined by paired t test. All data is represented as mean ±SEM from multiple independent experiments. ns: non-significant, *P < 0.05, **P < 0.01 and ***P < 0.001.

**Figure 4.**
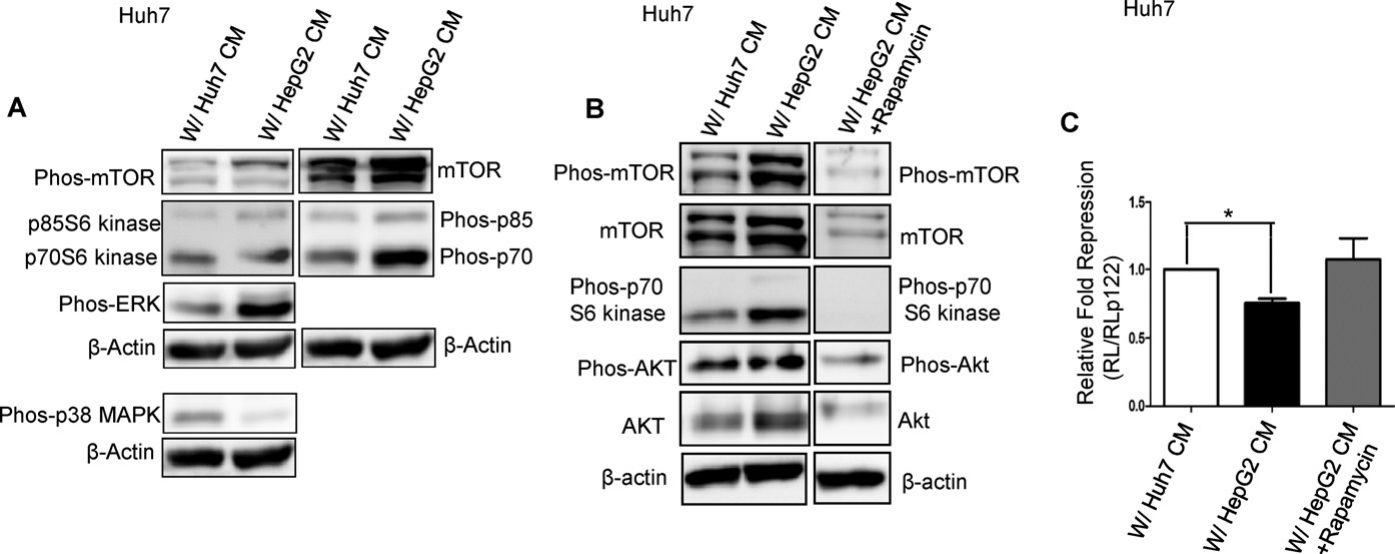
HepG2 CM activates mTOR pathway in Huh7 cells. (**A**) Western blot analysis was done to detect the levels of phosphorylated and nonphosphorylated mTOR and its downstream substrate kinases in Huh7 cells incubated with either HepG2 CM or Huh7 CM (control) for 48 h with a change after every 24 h. Also the levels of phosphorylated ERK and p38 MAPK were detected. (**B**) Effect of Rapamycin on phosphorylation of mTOR and its downstream kinases in Huh7 cells incubated with HepG2 CM in absence or in presence of 50 nM Rapamycin. Immunoblotting was done to detect the effect. (**C**) Effect of Rapamycin on miR-122 activity change in Huh7 cells transfected with RL reporters and incubated with HepG2 CM in presence or absence of 50 nM Rapamycin. After 48 h cells were lysed and luciferase activity was measured. Experiments were done in triplicate. Data are presented as means ±SEM of the Relative fold repression (control mean fold repression = 1) and significance is *P* = 0.0359.

**Figure 5.**
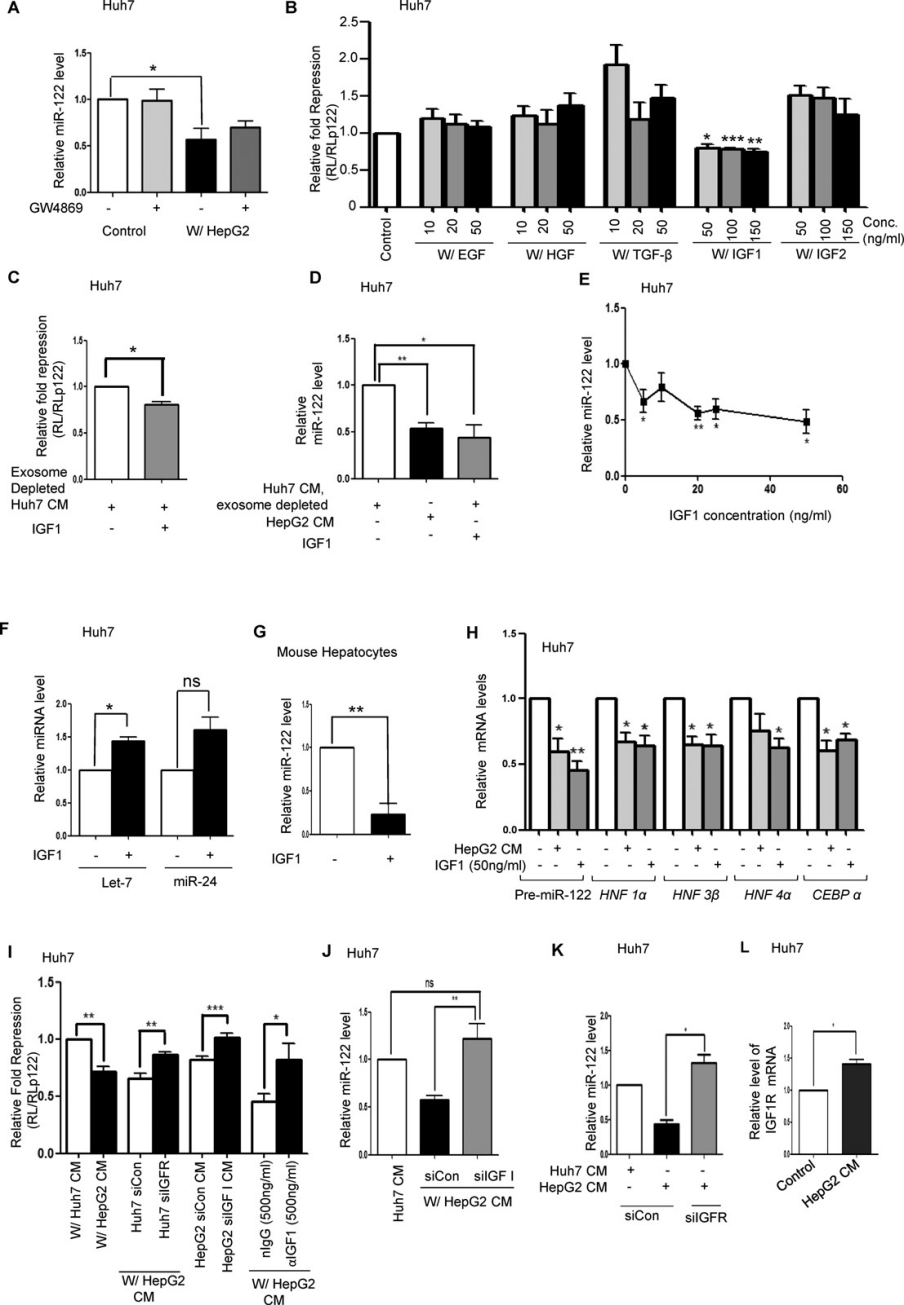
IGF1 secreted by HepG2 reduces activity and expression of miR-122 in Huh7 cells. (**A**) Effect of GW4869 on transfer of anti-miR-122 signal from HepG2 to Huh7 cells. HepG2 cells and Huh7 cells were either co-cultured together or mixed after being cultured separately for 48 h in presence and in absence of GW4869. Real-time quantification of miR-122 was then done to detect the level of miR-122 in both control and co-cultured samples in presence or absence of GW4869. Data represents three independent experiments with qPCR for each experiment being conducted in triplicate. *P* values were calculated by paired t test. (**B**) Effect of different growth factors on miR-122 activity in Huh7 cells. Huh7 cells transfected with RL reporters were incubated for indicated concentrations (ng/ml) of Epidermal Growth Factor (EGF), Hepatocyte Growth Factor (HGF), Transforming Growth Factor β (TGF-β), Insulin-like Growth Facto1 (IGF1) and Insulin-like Growth Factor 2 (IGF2) overnight in DMEM and luciferase activities were measured. Fold repression was estimated by dividing the normalized RL levels in RL-con and RL-per-miR-122 expressing cells. Relative fold repression was determined by setting the repression level of control as 1. (**C, D**) Effect of IGF1 on miR-122 activity (C) and Level (D) in Huh7 cells. Cells were incubated with exosome depleted Huh7 CM alone or supplemented with 100 ng/ml IGFI for 72 h with fresh changes after every 36 h. HepG2 CM was used as a positive control. (**E**) Dose response curve to determine the effect of various concentrations of recombinant IGF1 (ng/ml) on the miR-122 level of Huh7 cells. Huh7 cells were incubated for 24 h with DMEM containing IGF1 (0–50 ng/ml). Total RNA was extracted from the cells and qPCR was done to determine the miR-122 level. We found that the decrease in miR-122 level starts from 5 ng/ml of IGF1. For panel C experiments were performed in triplicate and *P* value was calculated by using unpaired t test. For panels D and E data represents four independent experiments with qPCR for each experiment being conducted in triplicate. *P* values were calculated by paired t test. (**F**) miR-24 and let-7a level change detected by real-time quantification in Huh7 cells treated with IGF1. Data represents three independent experiments with qPCR for each experiment being conducted in triplicate. *P* values were calculated by paired t test. (**G**) Effect of IGF1 on miR-122 level in primary mouse hepatocytes treated with IGF1. Data represents four independent experiments with qPCR for each experiment being conducted in triplicate. *P* values were calculated by paired t test. (**H**) Quantification of pre-miR-122, and other hepatic nuclear factor expression in Huh7 cells incubated for 72 h either with HepG2 CM or exosome depleted Huh7 CM containing 0 and 50 ng/ml of IGF1. Data represents four independent experiments with qPCR for each experiment being conducted in triplicate. *P* values were calculated by paired t test. (**I**) Effect of IGF1 depletion in HepG2 or IGF1R depletion in Huh7 on miR-122 activity in Huh7 cells in presence of HepG2 CM. Huh7 cells (control or IGF1R depleted), expressing miR-122 RL reporter, were incubated with CM from normal or IGF1 depleted HepG2 for 72 h to determine the specificity of IGF1 to decrease miR-122 activity in Huh7 cells. For control experiments, non-target siRNA was used. Incubation of HepG2 CM with αIGF1 antibody removed the anti-miR-122 activity. nIgG was used as a control. Fold repression was estimated by dividing the normalized RL levels in RL-con and RL-per-miR-122 expressing cells. Relative fold repression was determined by taking the control as 1 and expressing repression values relative to 1. Experiments were performed in triplicate and *P* value was calculated by using paired t test. (**J**) Effect of IGF1 depletion on miR-122 level in Huh7 cells incubated with CM from HepG2 cells transfected with a non-target or IGF1 specific siRNAs. The miR-122 level of the cells was detected by RT-PCR. Data represents five independent experiments with qPCR for each experiment being conducted in triplicate. *P* values were calculated by paired t test. (**K**) miR-122 level in Huh7 cells depleted for IGF1R (siIGF1R transfected) against control siRNA transfected cells in presence of HepG2 CM. Cellular miR-122 levels were quantified by RT-PCR. Data represents three independent experiments with qPCR for each experiment being conducted in triplicate. *P* values were calculated by paired t test. (**L**) Effect of HepG2 CM on IGFR1R expression in Huh7 cells. Huh7 cells incubated for 72 h with HepG2 CM were analysed for IGF1R mRNA levels by qRT-PCR. Normalization was done by 18S rRNA. Data represents three independent experiments with qPCR for each experiment being conducted in triplicate. *P* values were calculated by paired t test. All data is represented as mean ±SEM from multiple independent experiments. ns: non-significant, *P < 0.05, **P < 0.01 and ***P < 0.001.

### Statistical analysis

All graphs and statistical analyses were generated in GraphPad Prism 5.00 (GraphPad, San Diego, CA, USA). Nonparametric unpaired t-test and paired t test were used for analysis, and *P* values were determined. Error bars indicate mean ±SEM.

## RESULTS

### Intercellular transfer of miR-122 between human hepatic cells

To determine the importance of crosstalk between liver cells in the homeostasis of miRNA expression, we used human hepatoma cells HepG2 and Huh7 for our study. Despite its hepatic origin, HepG2 has highly reduced levels of miR-122 whereas Huh7 expresses this miRNA in high amounts (28,29). We wanted to see if miR-122 from Huh7 cells could be transferred to HepG2 cells in a co-culture model. HepG2 cells transfected with a plasmid encoding RL reporter with one perfect miR-122 binding site were co-cultured with Huh7 cells or, as a control, with non-transfected HepG2 cells. We documented increased repression of miR-122 reporter in HepG2 cells co-cultured with Huh7 cells where the extent of repression was determined by HepG2 to Huh7 cell ratio in the co-culture (Figure 1A–C).

To determine whether repression of miR-122 reporter in HepG2 is due to an actual transfer of miRNA between the co-cultured cells or due to an induction of miR-122 expression in HepG2 by Huh7 cells, GFP expressing HepG2 cells were co-cultured with DsRed expressing Huh7 cells for 48 h before they were sorted to GFP and dsRed positive cell pools (Figure 1D and Supplementary Figure S1). As a control, separately grown GFP positive HepG2 and DsRed positive Huh7 were mixed together just before sorting and also separated to individual pools. Relative quantification of the miR-122 levels in the sorted HepG2 cells that otherwise have low levels of mature miR-122 indicated a 9-fold increase of mature miR-122 level upon co-culture with Huh7 cells while the let-7a miRNA level in sorted HepG2 cells remained unchanged (Figure 1D and E). Interestingly, when measured, no corresponding increase in miR-122 precursor was detected in the sorted HepG2 cells (Figure 1F and G). These results indicate direct transfer of mature miR-122 to HepG2 cells grown in a co-culture with Huh7 cells.

Cyclin G1 is a target of miR-122. Previously, it was reported that miR-122 expression in HepG2 cells decreased Cyclin G1 expression and was accompanied by an increase in p53 protein (29). Upon co-culture with Huh7, western blotting of sorted HepG2 cells revealed a reduced level of Cyclin G1 protein as compared to control. This was associated with an increase in the p53 protein level (Figure 1H). Consequent real-time quantification of various miR-122 target mRNAs (32) such as *CAT1* (Solute Carrier Family 7 Member 1/Cationic Amino Acid Transporter 1)*, Aldolase* (Aldolase A), *GTF2B* (General Transcription Facor IIB) and *GYS1* (Glycogen Synthase 1) revealed a decrease in their expression in HepG2 cells co-cultured with Huh7 cells as compared to control (Figure 1I).

For intercellular transfer of miR-122 in hepatic cells, intercellular contacts are not required. Incubation of reporter transfected HepG2 cells with CM from Huh7 cells for 24 h also resulted in an increase in fold repression of miR-122 reporter in HepG2 cells (Figure 1J). This property of Huh7 CM was retained in the ≥100 KDa cutoff fraction. Northern analysis confirmed the transfer of mature miR-122 to HepG2 cells when incubated either with Huh7 CM or with its ≥100 KDa cutoff fraction. Exosomes, isolated from the ≥100 KDa cutoff fraction of Huh7 CM when added to HepG2 cells, were able to transfer miR-122 to the recipient cells. These results suggest transfer of mature form of miR-122 from Huh7 to HepG2 cells, possibly via exosomal vesicles (Figure 1J and Supplementary Figure S2). Secretion of miRNA containing exosomes is regulated by neutral Sphigomyelinase II (nSMNase II) gene in mammalian cells (15,33). This protein is known to hydrolyze sphingomyelins to generate ceramides and trigger the budding of exosomes. GW4869 is a specific inhibitor for neutral Sphingomyelinase II and decreases exosome secretion in mammalian cells (15). The secretion of miR-122 containing exosomes from Huh7 was found to be inhibited with increasing concentrations of GW4869, which also lowers the tetraspanin CD63 (34,35) and ALIX, two mammalian exosomal marker proteins (Figure 1K). These results are consistent with exosomes being the probable vehicle for intercellular miR-122 delivery. Confirming the importance of exosomal delivery in intercellular miR-122 transfer, the increase in miR-122 activity in HepG2 cells upon co-culture with Huh7 was prevented in the presence of GW4869 (Figure 1L). The notion that exosomal export of miRNA plays the key role in transfer of miR-122 from Huh7 to HepG2 cells was further confirmed when treatment of Huh7 cells with siRNAs against nSMNase II resulted in inhibition of miR-122 activity transfer from Huh7 cells to co-cultured HepG2 cells (Figure 1M).

Both Transmission and Scanning Electron micrographs (SEM and TEM) of Huh7 exosomes isolated by ultracentrifugation at 100 000xg confirmed the presence of vesicles of ∼100 nm in size in exosome preparation used for the assays (Supplementary Figure S3A). This was substantiated by dynamic light scattering analysis of exosomes that revealed bell-shaped curves with the peak of highest intensity at diameters of ∼100 nm (Supplementary Figure S3B). Affinity purified CD63 and CD81 (36) positive exosomes purified with Exo-FLOW^TM^ Exosome Purification Kit from ‘System Biosciences’ and stained with the lipophilic Exo-FITC universal exosome stain showed shifts in the FITC fluorescence upon binding with affinity beads (Supplementary Figure S3C and D). This confirmed the presence of exosomal markers in the Huh7 exosomes used in our study. Profiling of RNAs prepared from the exosomes also suggested enrichment of ‘exosomes’ in the preparations either isolated by exoquick kit or by ultracentrifugation (Supplementary Figure S3E).

Imaging of exosomes by Atomic Force Microscope at lower (Supplementary Figure S4A) and higher magnifications (Supplementary Figure S4B) revealed vesicular structures with clusters of aggregated particles. Profile analysis of cross sections revealed vesicle diameters of ∼100 nm. This was further substantiated with Nanoparticle Tracking analysis (NTA). Exosomes isolated by ultracentrifugation at 100 000xg were analysed at two concentrations: undiluted and at 1/10th dilution (Supplementary Figure S4C). FTLA analysis indicates a decrease in average diameter size with the increase in dilution that suggests presence of aggregates which are removed with increasing dilution (Supplementary Figure S4C).

### Exosomal transfer of miR-122 to human HepG2 cells leads to decreased growth and increased senescence

miR-122 overexpression in HepG2 cells decreased its’ growth rate along with an impairment in its invasion capability (29). To determine whether Huh7 co-cultured HepG2 cells show a similar phenotype, equal numbers of GFP positive HepG2 and DsRed expressing Huh7 cells were either co-cultured together or, as a control, seeded separately to have comparable confluency to that of the co-cultured cells. After 24, 48 and 72 h in culture, cells were collected by trypsinization and were paraformaldehyde fixed. As a control, separately seeded Huh7 and HepG2 cells were trypsinized, mixed together and were fixed. These were subjected to FACS analysis to score the percentage of GFP positive and DsRed positive cells in each sample. The relative growth for each cell was calculated by dividing the percentage of Green/Red cells in the co-cultured sets with that in the control sets. It was observed that the growth rate of HepG2 cells co-cultured with Huh7 decreased with time (relative growth rate of <1). Interestingly, the growth rate of Huh7 cells was increased in a co-culture with HepG2 (relative growth rate of >1) (Figure 2A). Thus, in a co-culture, HepG2 and Huh7 cells affect the growth of each other in a reciprocal manner. The effect of Huh7 on HepG2 growth is mediated via transfer of miR-122 as inhibition of miR-122 in Huh7 resulted in impaired growth retardation of neighbouring HepG2 cells (Supplementary Figure S5A).

The effect of Huh7 on HepG2 growth was further validated by immunofluorescence assays which score the Proliferating Cell Nuclear Antigen (PCNA) positive nuclei containing cells in a population. DsRed expressing HepG2 cells co-cultured with Huh7 cells showed decreased nuclear localization of PCNA as compared to control. However, the effect was reversed if the Huh7 used in co-culture was pre-transfected with anti-miR-122 oligonucleotides to block miR-122 activity, but not in the case of anti-let-7a oligonucleotides (Figure 2B and Supplementary Figure S6A).

Colony-forming ability of HepG2 cells, scored by number and size of colonies, is a marker of its proliferative potential. Cells pre-treated with exosomes isolated from Huh7 cells showed a reduced colony size compared to control. Exosomes from anti-miR-122 treated Huh7 cells also decreased colony formation of recipient HepG2 cells; however this decrease was less compared to control anti-let-7a transfected Huh7 exosomes. Anti-miR-122 treatment could not fully block the Huh7 exosomes’ effect on HepG2 colony size, possibly due to incomplete inhibition of miR-122 in transfected Huh7 cells (Figure 2C). This may have been because of the low transfection efficiency of Huh7. Hence, in an effort to induce miR-122 ‘loss of function’, anti-miR-122 and anti-let7 oligonucleotides were introduced in HepG2 cells which were then treated with Huh7 exosomes. Anti-miR-122 treatment but not the anti-let-7a treatment resulted in reversal in the reduction in colony size of HepG2 cells in the presence of Huh7 exosomes (Figure 2D). When grown in the presence of tritiated thymidine (^3^H), Huh7 exosome treated HepG2 showed reduced incorporation of ^3^H as compared to untreated control. Treatment of cells with exosomes isolated from miR-122 expressing HepG2 cells also reduced ^3^H incorporation in target HepG2 cells, signifying a reduced proliferation of the treated cells (Figure 2E). Confirming the importance of miR-122 in this effect, exosomes from Huh7 cells inhibited for miR-122 did not reduce ^3^H incorporation in HepG2 cells (Figure 2F).

HepG2 cells incubated with Huh7 exosomes also exhibited changes in other growth related properties. There was an increase in cellular senescence as demonstrated by increased β-galactosidase staining when HepG2 cells were treated with Huh7 exosomes. However, incubation with exosomes isolated from Huh7 cells transfected with anti-miR-122 oligonucleotides had little effect on the senescence status of treated HepG2 cells (Figure 2G and Supplementary Figure S6B). Transfection of donor Huh7 cells with anti-let-7a oligonucleotides did not affect the senescence induction capacity of Huh7 exosomes. Therefore, transfer of miR-122 from Huh7 to HepG2 cells results in decreased proliferation of HepG2 cells accompanied by increased senescence.

Doxorubicin is the most widely used drug in treatment of intermediate-advanced HCC (29,37). We attempted to determine the doxorubicin sensitivity of HepG2 cells when conditioned with miR-122 containing exosomes from Huh7 cells. Western blots revealed increased PARP and Caspase 9 cleavage in HepG2 cells treated with 50 μg/ml doxorubicin for 24 h in the presence of Huh7 exosomes. This was reversed in cells treated with exosomes isolated from Huh7 cells transfected with anti-miR-122 but not with anti-let-7a oligonucleotides. Thus treatment of HepG2 with miR-122 containing exosomes from Huh7 cells leads to increased apoptosis when challenged with doxorubicin (Figure 2H). Similar to that isolated with ultracentrifugation methods, Huh7 exosomes isolated with Exo-FLOW^TM^ Exosome Purification Kit from ‘System Biosciences’ also showed growth retardation and drug sensitivity related effects on treated HepG2 cells (Supplementary Figure S7).

### Reciprocal inhibition of miR-122 expression in neighbouring cells by the human HCC cell HepG2

Huh7 is a hepatoma cell that exhibits constitutive expression of miR-122 which is associated with low Cyclin G1 expression level in hepatic cells (29,38,39). Previous experiments indicated that in the presence of HepG2, growth rate of Huh7 increases (Figure 2A). Does HepG2 reduce miR-122 in neighbouring Huh7 cells to increase their proliferation? To investigate this further, Huh7 cells expressing miR-122 reporter were co-cultured either with non-transfected control Huh7 cells or HepG2 cells at a ratio of 1:1. In the presence of HepG2, there was a decrease in the relative fold repression of miR-122 reporter in Huh7 cells as compared to control (Figure 3A and B). Quantitative PCR indicated that there was a decrease in the miR-122 level in HepG2 co-cultured, as opposed to the control Huh7 cells (Figure 3C). This tells us that the observed decrease in miR-122 activity is because of a reduced miR-122 level in Huh7 cells co-cultured with HepG2. Real-time quantification further revealed a similar change in the precursor form of miR-122 (pre-miR-122) in HepG2 exposed Huh7 cells, suggesting a reduced production of miR-122 (Figure 3D). This decrease in transcription appears to be specific for the miR-122 promoter and does not depend on miR-122 identity. When Huh7 cells were transfected with plasmids expressing pre-miR-122 under a U6 promoter (31), there was no change in the miR-122 level in control and HepG2 co-cultured Huh7 cells (Figure 3E). Therefore, it may be concluded that HepG2 cells exert a reciprocal effect on the miR-122 level in co-cultured Huh7 cells primarily by reducing the production of pre-miR-122.

How does HepG2 reduce pre-miR-122 expression in Huh7 cells? Huh7 cells transfected with miR-122 reporter showed a decreased repression even when they were incubated with CM from confluent HepG2 cells (Figure 3F). Real-time quantification in HepG2 CM treated Huh7 cells confirmed a decreased level of cellular miR-122 (Figure 3G). HepG2 CM was also effective in downregulating pre-miR-122 expression in Huh7 cells (Figure 3H).

In hepatic cells, expression of miR-122 is controlled primarily by four liver enriched transcription factors (LETFs) (25,36,40). We wanted to check the expression level of the LETFs – HNF 1α, HNF 3β, HNF 4α and C/EBP α in Huh7 cells incubated with HepG2 CM. This was done to verify whether the decrease of miR-122 in Huh7 cells by HepG2 was because of a decreased expression of LETFs. Low miR-122 level in hepatic tumours correlates with reduced expression of these liver-specific transcription factors, suggesting a regulatory role for these proteins on hepatic miR-122 level (19,25,40). Real-time analysis confirmed reduced expression of several of these LETFs accompanied by reduced miR-122 expression in Huh7 cells treated with HepG2 CM (Figure 3I). CHIP-PCR analysis using antibodies against RNA polymerase II, HNF1α, HNF3β and HNF4α (36,41) revealed reduced binding of the miR-122 promoter element by these transcription factors in Huh7 cells treated with HepG2 CM (Figure 3J). Therefore, it is evident that factor(s) present in HepG2 CM are able to cause reduction in the levels of miR-122 and pre-miR-122 in Huh7 cells by reducing LETF activity and their binding to miR-122 promoter.

### HepG2 secreted factors increase mTOR signalling in neighbouring hepatic cells

Downregulation of miR-122 is a characteristic feature of human HCC and this is accompanied with the deregulation of mTOR signalling which plays a pivotal role in the pathogenesis of HCC (42–44). There is also evidence that the p38 MAPK cascade is downregulated in human HCCs that accounts, in part, for the resistance of cells to apoptosis, leading to unrestricted growth of human HCCs (45,46). Also, the activity of ERK1/2 is significantly higher in human HCCs than in adjacent nontumorous lesions (46). Huh7 cells in the presence of HepG2 CM demonstrate reduced miR-122 expression along with an increased growth rate (Figure 2A) To identify the nature of the factor responsible for downregulation of miR-122 in Huh7 cells, the status of different intracellular signals in Huh7 cells incubated with HepG2 CM was checked. As a control, Huh7 cells were incubated for the same duration with Huh7 CM. Upon incubation with HepG2 CM, there was an increase in the phosphorylation of mTOR with respect to control. This increase was coupled with an elevation in the levels of phosphor-p70 S6 kinase, a downstream target of mTOR. On the other hand, phosphorylated p38 MAPK levels appeared to decrease in HepG2 treated Huh7 cells as compared to control. There was also an increase in the level of phosphorylated ERK1/2 (Figure 4A). This increase in the phosphorylated form of various proteins of the AKT/mTOR pathway was found to be reversed in the presence of 50 nM of Rapamycin, the mTOR inhibitor (Figure 4B). In the presence of Rapamycin, the HepG2 CM induced decreased miR-122 activity in Huh7 was also found to be reversed thereby indicating that the decreased expression of miR-122 in Huh7 cells in the presence of HepG2 is mediated by the Rapamycin sensitive AKT/mTOR signalling pathway (Figure 4C).

### HepG2 secreted IGF1 reduces miR-122 expression and activity in neighbouring cells

HepG2 cells secrete factors that activate AKT/mTOR and ERK signalling pathways in the neighbouring Huh7 cells, concomitant with a corresponding decrease in miR-122 expression. We attempted to decipher the nature of this secreted factor. To determine whether this secreted factor was exosomal, Huh7 cells were co-cultured with HepG2 cells in the presence or absence of GW4869. No effect of the drug on the ability of HepG2 secreted factor to reduce miR-122 level in Huh7 cells was noticed (Figure 5A). Thus the factor secreted by HepG2 cells, which induces a decrease in miR-122 expression in Huh7 cells, is likely non-exosomal.

HCC cells are known to express various growth factors such as Epidermal Growth Factor (EGF), Hepatocyte Growth Factor (HGF), Transforming Growth Factor β (TGF-β) and Insulin-like Growth Factor (IGF), which induce cell proliferation in an autocrine fashion (47). The receptors of these growth factors are known to activate intracellular signals such as the Raf/MEK/ERK pathway and the PI3K/AKT/mTOR pathway, which induce proliferation of HCC cells (48). We incubated miR-122 reporter transfected Huh7 cells with DMEM containing various growth factors for 16 h. Luciferase assays revealed that various concentrations of IGF1 when added to DMEM lead to a decrease in miR-122 activity in Huh7 cells as compared to control and other growth factor treated Huh7 cells (Figure 5B). Quantification revealed a corresponding decrease in miR-122 level and activity upon IGF1 treatment of Huh7 cells (Figure 5C, D and E). No such decrease was observed for two other miRNAs tested in IGF1 treated Huh7 cells, confirming the miRNA specificity of IGF1 (Figure 5F). Similar decrease of miR-122 was also noted in mouse primary hepatocytes incubated with IGF 1 in culture (Figure 5G). Pre-miR-122 and mRNA levels of various Hepatic Nuclear Factors like HNF1α, HNF3β, HNF4α and CEBPα were quantified in Huh7 cells treated with IGF1 (Figure 5H). In the presence of IGF1, Huh7 cells showed decreased pre-miR-122 and lower levels of the HNFs. This is consistent with the notion that IGF1 inhibits miR-122 expression in Huh7 cells.

Knockdown of Insulin Like Growth Factor 1 Receptor in Huh7 cells by siRNAs resulted in a reversal of the decrease in both miR-122 activity and level upon incubation with HepG2 CM. CM from siIGF1 transfected HepG2, in contrast to siControl transfected HepG2, was unable to reduce neither miR-122 activity or level in target Huh7 cells (Figure 5I and J). It also failed to induce proliferation of neighbouring Huh7 cells in a co-culture of siIGF1 transfected GFP positive HepG2 and untransfected DsRed expressing Huh7 (Supplementary Figure S5B). Similarly, depletion of IGF1R in Huh7 cells made them immune to HepG2 CM mediated downregulation of miR-122 activity and level in Huh7 cells (Figure 5I and K). When IGF1-antibody blocked HepG2 CM was added, there was no inhibitory effect associated with HepG2 CM on miR-122 activity in Huh7 cells; rather an increase in miRNA activity was observed (Figure 5I). Therefore, as compared to nIgG, α-IGF1 blocked CM showed increased miR-122 activity, suggesting that IGF1 present in HepG2 CM causes inhibition of miR-122 expression leading to decreased miR-122 activity in Huh7 cells. Interestingly, treatment with HepG2 CM increases IGF1R expression in target Huh7 cells. IGF1R is a known target of miR-122 (28) and hence this increase may possibly be a consequence of the decreased miR-122 expression in Huh7 cells (Figure 5L).

### HepG2 cells overcome tumour suppressing effect of exosomal miR-122 by secreting IGF1

**Figure 6.**
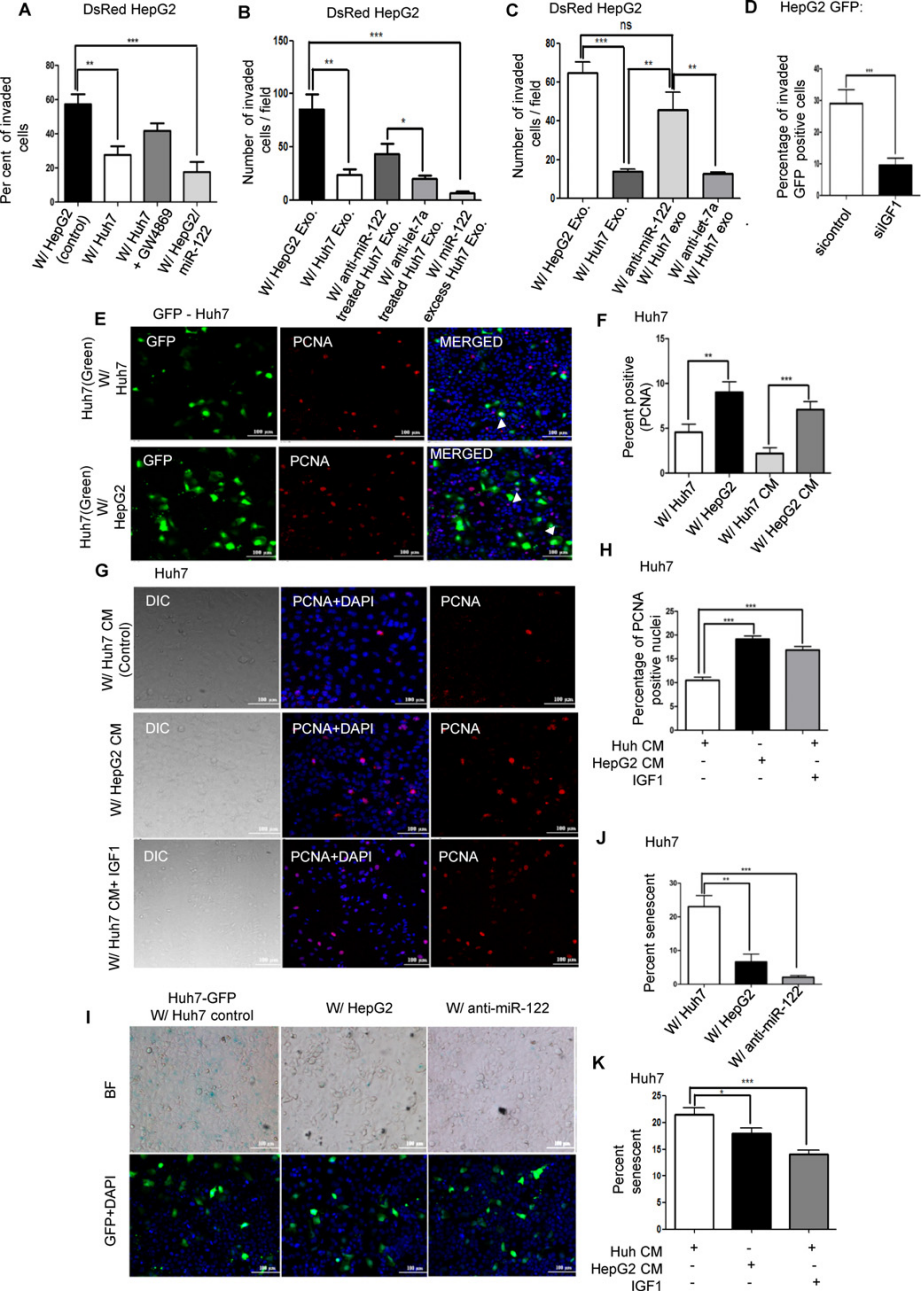
HepG2 cells overcome the tumour suppressive effect of miR-122 containing exosomes by secreting IGF1. (**A**) Effect of co-culture with miR-122 expressing cells on matrigel invasion properties of HepG2. DsRed expressing HepG2 were co-cultured with HepG2 (control) or Huh7 cells (with or without GW4869 treatment) on matrigel coated polycarbonate membrane having a pore size of 8 μm. After 48 h, cells were fixed, and the number of cells which had invaded through the matrigel layer to the outer side was determined. HepG2 cells expressing miR-122 was used as a positive control. DAPI stained the nucleus and the percentage of invaded DsRed cells was determined by counting the number of DAPI and DsRed double positive cells. Results represent data from ≥ 5 independent fields taken from two independent experiments. (**B**) Effect of miR-122 containing exosome treatment on matrigel invasion properties of HepG2 cells. HepG2 cells stably expressing DsRed were incubated with exosomes isolated either from HepG2, Huh7, anti-miR-122 or let-7a expressing Huh7, or from Huh7 exogenously expressing miR-122. The number of DsRed cells invaded through matrigel was counted. (**C**) Experiments similar to described in B were done with HepG2 cells pre-transfected with anti-miR-122 and anti-let-7a oligos. Results represent data from ≥6 independent fields taken from two independent experiments. (**D**) Effect of IGF1 depletion on invasive property of HepG2 co-cultured with miR-122 expressing Huh7 cells. HepG2 cells expressing GFP were transfected either with siRNAs against IGF1 or with a control siRNA. Cells were seeded onto Huh7 cells layered on matrigel coated polycarbonate membranes having pore sizes of 8 μm. The number of invaded GFP positive cells colocalizing with DAPI was counted after 24 h of co-culture. Results represent data from ≥15 independent fields taken from two independent experiments. (**E, F**) Effect of HepG2 co-culture or HepG2 CM treatment on proliferation of Huh7 cells. GFP transfected Huh7 cells were co-cultured with nontransfected Huh7 (control) or HepG2 cells for 72 h. After 72 h, cells were fixed and immunofluorescence detection of PCNA (mitosin) was done in panel E. The percentage of PCNA positive nuclei (red) colocalizing with GFP (green) was calculated. Results represent data from eight independent fields taken from two independent experiments. The experiment was also done for GFP transfected Huh7 cells incubated with Huh7 CM (control) and HepG2 CM for 72 h. *n* = 7 independent fields taken from two independent experiments. (**G, H**) Effect of IGF1 treatment on proliferation of Huh7 cells. Huh7 cells were grown with HepG2 CM and exosome deprived CM from Huh7 cell cultures (with and without added IGFI) and the number of PCNA positive cells was determined. Results represent data from ≥10 independent fields taken from two independent experiments. (**I, J**) Senescence status of GFP positive Huh7 cells co-cultured with non-transfected Huh7 cells (control), HepG2 cells and Huh7 transfected with anti-miR-122 for 72 h. Cells were then fixed and senescent cells detected by β-galactosidase staining. The number of senescent GFP positive cells was then counted and the percentage calculated. Results represent data from ≥ 6 independent fields. (**K**) Change in senescence in Huh7 cells after IGF1 exposure. Huh7 cells were incubated with CM from HepG2, exosome depleted CM from Huh7 with or without 100 ng/ml of IGF1. After 72 h β-galactosidase activity was detected and the percentage of senescent cells was calculated. Results represent data from ≥10 independent fields from two independent experiments. All data are presented as mean ±SEM and *P < 0.05, **P < 0.01 and ***P < 0.001. *P* value was calculated by unpaired t test.

As evidenced from the individual growth rate curves of mixed cell populations described earlier in Figure 3A, it seems that when co-cultured, HepG2 and Huh7 cells reciprocally regulate each other's growth. miR-122 is known to repress genes involved in metastasis, invasion (ADAM10, ADAM17) and apoptosis (Bcl-w) (26,28,49). Hence, we hypothesized that co-culture of Huh7 with HepG2 cells would lead to reduced invasion of HepG2 due to transfer of miR-122 from Huh7. We co-cultured stably transfected DsRed positive HepG2 cells with Huh7 and determined the percentage of invaded HepG2 cells on matrigel. In the presence of Huh7, HepG2 cells showed reduced invasion. In the presence of GW4869 which inhibits exosome release, the effect of Huh7 on HepG2 invasion was reduced and higher number of HepG2 invaded the matrigel. As expected, co-culture of DsRed HepG2 with HepG2 overexpressing miR-122 leads to a reduced number of DsRed cells invading through the matrigel (Figure 6A and Supplementary Figure S8). Exosomes from Huh7 also reduced the invasive ability of HepG2 cells. Huh7 exogeneously expressing pre-miR-122 leads to a lesser number of DsRed HepG2 cells invading through matrigel than control (Figure 6B and Supplementary Figure S9A). Addition of exosomes from Huh7 transfected with anti-miR-122 oligonucleotides only partially reversed the effect of Huh7 exosomes. This may have been because of the reduced transfection efficiency of Huh7 cells which resulted in incomplete inhibition of transferable miR-122 via exosomes. However, anti-miR-122 oligonucleotides transfected HepG2 cells treated with Huh7 exosomes resulted in complete reversal of the reduction in invasive potential observed in the case of anti-let-7a transfected HepG2 cells (Figure 6C and Supplementary Figure S9B). These experiments demonstrate the tumour suppressive and anti-invasive activity of the transferred miR-122 on target HepG2 cells.

IGF1 secreted by HepG2 inhibits miR-122 biogenesis in co-cultured Huh7 cells, and this in turn may be the reason behind the increased growth rate of Huh7 observed in HepG2 co-cultured cell populations. Huh7 cells in turn exert a restorative effect on HepG2 by transferring miR-122 to HepG2. Does the secretion of IGF1 from HepG2 have any effect on this restorative property of Huh7? Can HepG2 cells circumvent the growth inhibitory effect exerted by the transferred miR-122 by secreting IGF1? HepG2 cells expressing GFP were transfected with siRNAs against IGF1 or a control siRNA and were layered on Huh7 cells pre-seeded on matrigel. The percentage of invaded GFP positive cells was detected. HepG2 cells with compromised IGF1 (siIGF1 transfected) showed reduced invasion through Huh7 layered on matrigel than the control siRNA transfected HepG2 cells (Figure 6D and Supplementary Figure S9C). Thus, IGF1 released by HepG2 cells enabled the cells to become more invasive in nature through a layer of cells expressing miR-122.

### IGF1 increases proliferation of Huh7 cells

We next examined the phenotypic effect of IGF1 secretion by HepG2 on Huh7 proliferation. Huh7 cells expressing GFP were co-cultured with non-transfected Huh7 (control) or HepG2 cells and the percentage of PCNA and GFP-positve Huh7 cells co-localizing with DAPI was counted. In the presence of HepG2, Huh7 cells showed greater number of PCNA positive cells than control. This effect was replicated after incubation of Huh7 cells with HepG2 CM (Figure 6E and F). To determine the role of the secreted IGF1 in this phenomenon, we incubated GFP transfected Huh7 cells with exosome depleted CM from Huh7 (control), HepG2 or exosome depleted Huh7 CM containing 100 ng/ml of IGF1. In the presence of IGF1, Huh7 cells showed increased proliferation as evidenced by the percentage of PCNA positive GFP cells co-localizing with DAPI (Figure 6G and H).

Co-culture with HepG2 leads to reduced number of senescent Huh7 cells. The decrease in senescent cell number was also there after anti-miR-122 expression in Huh7 indicating that the inhibition of miR-122 expression by HepG2 could lead to reduced senescence of Huh7 cells (Figure 6I and J). HepG2 CM incubated Huh7 cells also exhibited reduced β-galactosidase staining that suggests less senescence in presence of an inhibitory factor secreted by HepG2 cells. The addition of 100 ng/ml of IGF1 reduced the number of senescent Huh7 cells. Thus IGF1, secreted by HepG2, could be responsible for the decrease in senescence in HepG2 CM treated or HepG2 co-cultured Huh7 cells (Figure 6K and Supplementary Figure S10).

## DISCUSSION

In this study, we have established a model in which cells having different miRNA profiles when co-cultured together can reciprocally regulate each other's miRNA levels, thereby affecting proliferation and senescence status of both cells. Intercellular transfer of miRNAs between cancer and normal cells has already been reported. Tumour suppressor miRNAs are usually downregulated in cancer cells, leading to tumour progression and metastasis. Past reports along with the results of this study indicate that cells with downregulated miRNAs are compensated by the transfer of exosomes from surrounding cells containing the decreased miRNAs. However, this method of maintaining homeostasis does not ultimately stop the progression of cancer. Hence, cancer cells must have developed a mechanism by which they can override this suppressive effect. We wanted to examine this crosstalk between cells and to that purpose developed a co-culture system involving hepatic cells having differential expression of the tumour suppressor miR-122. This miRNA is detected in very low amount in HepG2, whereas its expression in Huh7 cells is relatively abundant (28,29).

Here we show the exosome mediated transfer of miR-122 from Huh7 to HepG2 cells. The transferred miRNA was used in gene repression and could change the expression of various miR-122 regulated physiological genes in the recipient cells. Co-culture with HepG2 also leads to a reciprocal decrease in miR-122 level in Huh7 cells. This decrease appeared to be CM mediated but surprisingly was not exosome driven. Investigation of the signalling pathway involved revealed that incubation with HepG2 CM leads to an activation of the mTOR signalling pathway indicating the possible role of a growth factor in the process. Addition of various growth factors known to be secreted by HepG2 cells and other HCC cells lead to the identification of IGF1 as the candidate growth factor. Antibody inhibition experiments coupled with siRNA mediated knockdown of IGF1R in Huh7 cell and IGF1 in HepG2 cells proved the primary involvement of this factor in reduction of miR-122 in Huh7 cells and also in mouse primary hepatocytes.

Addition of Huh7 exosomes resulted in decreased growth and invasion of the recipient HepG2 cells. This decrease was only partially reversed when exosomes isolated from anti-miR-122 oligonucleotide transfected Huh7 cells were added to HepG2 cells. To determine the efficacy of the anti-miR-122 transfection in Huh7 cells, Huh7 cells transfected with anti-miR-122 oligonucleotides were analysed for cellular miR-122 levels (Supplementary Figure S11A and B). As a control Huh7 cells transfected with LNA^TM^ modified -anti-miR-122 and LNA^TM^-modified anti-miR-128 (control) oligonucleotides were similarly analysed. This was done based on previous reports that Locked Nucleic Acids modified (LNA) antisense oligonucleotides are more effective than standard 2’-*O*-methyl modified oligonucleotides in binding and inhibiting miRNA action in primate, human and rat liver cells (32,50). 2’-OMe-anti-miR-122 transfected Huh7 cells showed reduced levels (∼50%) of intracellular miR-122. This reduction was comparable to that obtained with LNA-anti-miR-122 oligonucleotides. Further decrease in miR-122 levels may not have been possible because of the low transfection efficiency of Huh7 cells which was determined to be ∼20% (Supplementary figure S11C).

Reduced levels of miR-122 lead to reduced repression of miR-122 reporters in luciferase assays (Supplementary Figure S11D). Also Huh7 cells transfected with anti-miR-122 oligos have higher levels of miR-122 targets compared to control (Supplementary Figure S11E and F). The reduced level of intracellular miR-122 is also translated into reduced levels of exosomal miR-122 released from anti-miR-122 oligonucleotide (both 2’-*O*-methyl and LNA modified) transfected cells (Supplementary figure S11G and H). However, anti-miR-122 treatment does not totally reduce miR-122 in exosomes. It may be argued that the remaining residual exosomal miR-122 has an effect on the recipient HepG2 cells and thus cause only partial reversal of the reduction in colony formation and invasion as shown in Figures 2C and 6B. Also, Figure 2C describes an experiment where HepG2 cells are incubated with exosomes from Huh7 for 7 days with changes after every 48 h. It may be hypothesized that the residual exosomal miR-122 in Huh7 transfected with anti-miR-122 are transferred to HepG2 during the longer incubation time involved and restore miR-122 level to an extent sufficient to observe the effect.

The IGF axis is a complex signalling network that is involved in many physiological and pathological processes such as mitogenesis, angiogenesis, transformation, differentiation, tissue homeostasis, anti-apoptosis and cell motility (51). Alterations in this signalling axis have been described in human hepatocarcinogenesis. In HCC, a decreased tissue expression of IGF2 and an increased expression of IGF1 receptor (IGF1R) have been reported (52). However, elevations in the serum level of IGF1 are correlated with an increased risk for developing breast, colon, prostrate and lung cancer in mouse models (53–55). Thus the role of IGF1 in the development of hepatocarcinogenesis, at least in the initial stages, cannot be ruled out.

The key molecules of this network are the peptide-ligands IGF1 and IGF2 and the receptors IGF1R, IGF2R and insulin receptor (INSR) (56). The IGF1R binds IGF1 and IGF2 with high affinity and insulin with very low affinity, whereas the INSR binds insulin with high affinity.

IGF1R and INSR activate multiple signalling cascades upon ligand binding. The pathways activated are mainly the Ras-Raf-ERK signalling pathway and the phosphatidylinositol-3-kinase/AKT (PI3K/AKT) signalling pathway. These pathways in turn activate multiple signalling cascades affecting cell proliferation, anti-apoptosis, differentiation, tissue homeostasis (Ras-Raf-ERK) as well as cell survival, metabolic actions, anti-apoptosis and differentiation (PI3K/AKT) (57). IGF1R is found overexpressed in many types of cancer cells and is upregulated in primary human HCCs. It has a miR-122 binding site in its 3’UTR and was shown by Bai *et al.* to be a target of miR-122. miR-122 and *Igf1r* were found to be reciprocally regulated in primary human HCCs (28).

Huh7 cells incubated with HepG2 CM show downregulated expression of miR-122. This is accompanied by an upregulation of IGF1R mRNA. This may serve as a positive feedback mechanism in which the IGF1 induced downregulation of miR-122 is further enhanced because of the accompanying upregulation of IGF1R. Thus, we may hypothesize that IGF1 secreted by HCC cells serves to downregulate miR-122 in the surrounding normal cells and causes tumour progression. This study illustrates a mechanism by which cancer progresses and reinforces the suitability of IGF1R as a therapeutic potential in HCC. Interestingly, IGF1 expression in HepG2 cells is decreased upon treatment with miR-122 containing Huh7 exosomes. In this context, there seems to be a reciprocal relationship between miR-122 and IGF1 expression (Supplementary Figure S12).

The *in vivo* situation in this context is yet to be determined. It would be interesting to speculate that the exosomal delivery of miR-122 containing exosomes between hepatic cells in liver tissue may serve as a mechanism for maintaining homeostasis of tissue miRNA. Various environmental and genetic factors contribute to HCC development. Hepatocarcinogenesis is a step wise process during which multiple genes are altered. The unbalanced expression of miR-122 during the initial steps of liver cancer development may be compensated by exosomal transfer of miR-122 from normal to cancer cells. However, upregulation of growth factors and their receptors, like IGF1R, may contribute to crossing the final hurdle towards cancer development. Additionally, miR-122-mediated downregulation of IGF1 release by hepatic cells may serve as another potential therapeutic avenue.

## SUPPLEMENTARY DATA

Supplementary Data are available at NAR Online.

SUPPLEMENTARY DATA
