# Supplementary material for: Insulin-like growth factor-1 prevents miR-122 production in neighbouring cells to curtail its intercellular transfer to ensure proliferation of human hepatoma cells
Source: Nucleic Acids Res. 2014 May 9;42(11):7170–85. doi: 10.1093/nar/gku346 (PMC4066773; doi:10.1093/nar/gku346)
Supplement: SUPPLEMENTARY DATA [file supp_42_11_7170__index.html]

SUPPLEMENTARY DATA 

# Insulin-like growth factor-1 prevents miR-122 production in neighbouring cells to curtail its intercellular transfer to ensure proliferation of human hepatoma cells

## SUPPLEMENTARY DATA

**Files in this Data Supplement:**

- Supplementary Data
