# Supplementary material for: Insulin-like growth factor-1 prevents miR-122 production in neighbouring cells to curtail its intercellular transfer to ensure proliferation of human hepatoma cells
Source: Nucleic Acids Res. 2014 May 9;42(11):7170–85. doi: 10.1093/nar/gku346 (PMC4066773; doi:10.1093/nar/gku346)
Supplement: SUPPLEMENTARY DATA [file supp_gku346_nar-01831-y-2013-File008.pdf]

Supplemental Materials

Table S1

List of Plasmids used

**Name of the Plasmid**	**Reference/Source**	**Plasmid Description**
pRL-Con	From Witold Filipowicz (1)	Humanized Renilla Luciferase (RL) coding region
pRL-Per-miR-122	Described earlier (2)	Perfect miR-122 binding site downstream of RL coding region
PGL3-FF	From Promega	Firefly Luciferase (FL) under SV40 Promoter.
pGFP	-	Plasmid expressing GFP protein, amplified and cloned in pCI neo vector
pDsRed-Monomer-N1	From Clontech	Plasmid expressing DsRed monomer fluorescent protein from CMV promoter
pmiR122	Described earlier (2)	Plasmid encoding pre-miR122 under a constitutive U6 promoter.

Table S2

List of siRNAs used

siRNA (Neutral Sphingomyelinase 2) SMPD2	L-006677-01-0005 Dharmacon	ON-TARGETplus SMARTpool - Human SMPD2
Human IGF1R	L-003012-00-0005 Thermo Scientific	ON-TARGETplus SMARTpool-Human IGF1R (3480)
Human IGF1	L-003011-00-0005 Thermo Scientific	ON-TARGETplus SMARTpool-Human IGF1 (3479)
SiControl	siRNA sequence not targeting any nuclear encoded genes	-------

Table S3

List of Primers

Primer	Forward Primer sequence (5'-3')	Reverse primer sequence (5'-3')
Human Aldolase	TGGACCTAGCTTGGCGCGGA	CCTGGGCCAGCAGGCAGTT C
Human GYS1F	GGTGGCTAACAAGGTGGGTGG	CGATCAGCCAGCGCCCGAAA
Human GTF2B	TGCGCGTCTCTTCGCCACAT	AGCTCCTGTGCCCTTGCCAAT
CAT1	GCCGCCGGCTTGGATTCTGA	CCCCGAGGGCCACCAGATCA
RL	CCAAGCAAGATCATGC	GCTCTTGATGTACTTACCC
Human IGF1R	CCGTGCGCTGGATGTCTCCTGAG	CGCCCTCCATGACGAAGCGAAGG
Human HNF1α	CTATGAGAGGCAGAAGAACC	CGTGTCCATGGCCAGCTTG
Human HNF3β	GGGCTCTGTCCGCAGGGC	CGCGCCGGGGGACATCC
Human HNF4α	GCAGGCTCAAGAAATGCTTC	GGACTCACACACATCTGCG
Human C/EBPα	GACCGCCCTGGGCCAGC	CTCGCAGGGAGAAGCCACC
Pre-miR-122	CCTTAGCAGAGCTGTGGAG	GCCTAGCAGTAGCTATTTAG
18S rRNA	TGACTCTAGATAACCTCGGG	GACTCATTCCAATTACAGGG
miR-122 promoter	TGACCAAAGGTGGTGCTGAC	TCACCTGCAGTCGAGGCTAA

Table S4

List of Antibodies used

**Name of Antigen**	**Raised in**	**Source**	**Dilutions used for Western Blot**
Human Cyclin G1	Mouse	Santa Cruz	1:500
Human p53	Rabbit	Biovision	1:1000
Cleaved PARP	Rabbit	Cell Signaling	1:1000
Cleaved Caspase 9	Rabbit	Cell Signaling	1:1000
Human Alix	Mouse	Santa Cruz	1:1000
Phospho mTOR	Rabbit	Cell Signaling	1:1000
mTOR	Rabbit	Cell Signaling	1:1000
Phos Akt	Rabbit	Cell Signaling	1:1000
Akt	Rabbit	Cell Signaling	1:1000
Phos p70/85 S6 kinase	Rabbit	Cell Signaling	1:1000
P70/85 S6 kinase	Rabbit	Cell Signaling	1:1000
Phos p38 MAPK	Rabbit	Cell Signaling	1:1000
Phos erk	Rabbit	Cell Signaling	1:1000
Mitosin	Rabbit	Bethyl	1:100 (IF)
HA	Rat	Roche	1:1000
Human IGF-1 (SAB3600001)	Goat	Sigma-Aldrich	For neutralization
nIgG	Rat	Calbiochem	-
β-actin	Mouse	Sigma-Aldrich	1:10,000
HNF3β	Rabbit	Cell Signaling	(CHIP) 1:25
HNF1∝	Rabbit	Bethyl Laboratories	(CHIP) 1:25
HNF4∝	Mouse	abcam	(CHIP) 1:25
RNA Polymerase II	Mouse	Santa Cruz Biotechnology	(CHIP) 1:50
CD63	Mouse	BD Pharmingen	1:1000

Supplementary figures

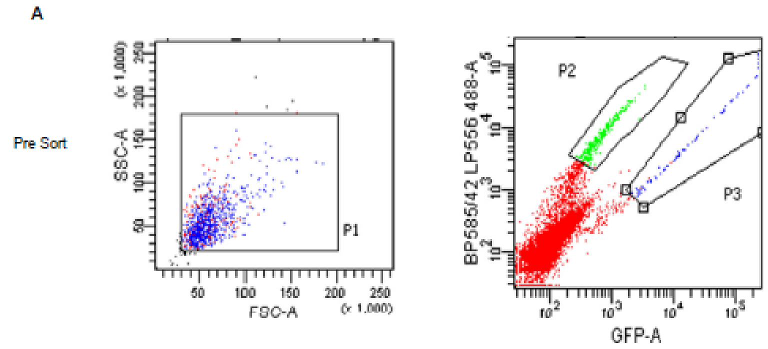

Population	#Events	%Parent	%Total
All Events	57,681	####	100.0
P1	54,111	93.8	93.8
P2 (GFP-HepG2)	1,570	2.9	2.7
P3 (DsRed-Huh7)	396	0.7	0.7

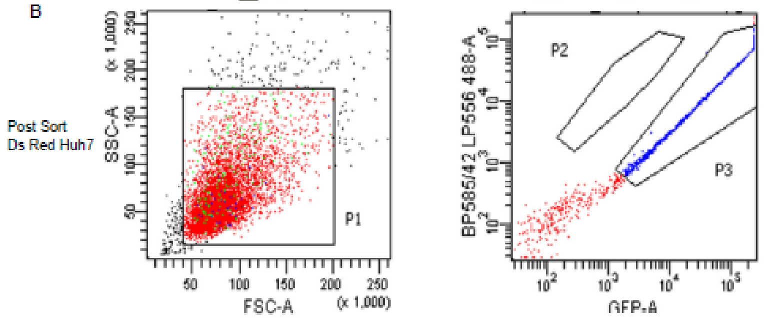

Population	#Events	%Parent	%Total
All Events	1,100	####	100.0
P1	1,062	96.5	96.5
P2 (GFP-HepG2)	0	0.0	0.0
P3 (DsRed-Huh7)	769	72.4	69.9

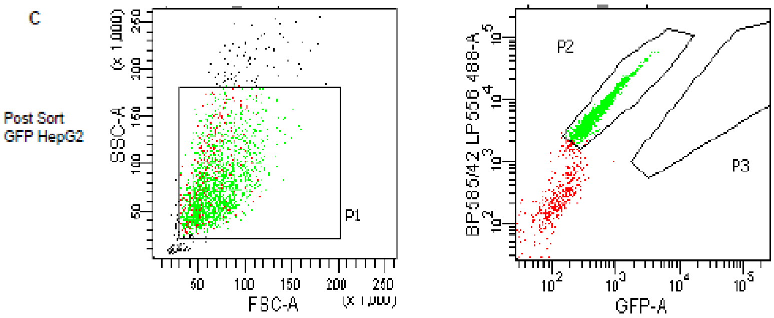

Population	#Events	%Parent	%Total
All Events	2,079	####	100.0
P1	1,904	91.6	91.6
P2 (GFP-HepG2)	1,512	79.4	72.7
P3 (DsRed-Huh7)	0	0.0	0.0

Figure S1 Distribution of GFP HepG2 and DsRed Huh7 cells in a population before and after FACS sorting. HepG2 cells expressing GFP were cocultured with DsRed expressing Huh7 cells for 48 h. **(A)** The Pre-sort images taken during FACS analysis showed a mixed cell population. Cells which had either green (blue) or red (green) fluorescence were sorted to separate pools and subsequently reanalysed. **(B-C)** The post-sort images of the sorted GFP HepG2 (panel B) and sorted DsRed Huh7 (panel C) are shown to confirm the purity. P1 represents the population of cells that were analysed, P2 represents DsRed positive cells and P3 stands for the GFP positive cell populations. The percentage of each cell in the population is shown below the respective images.

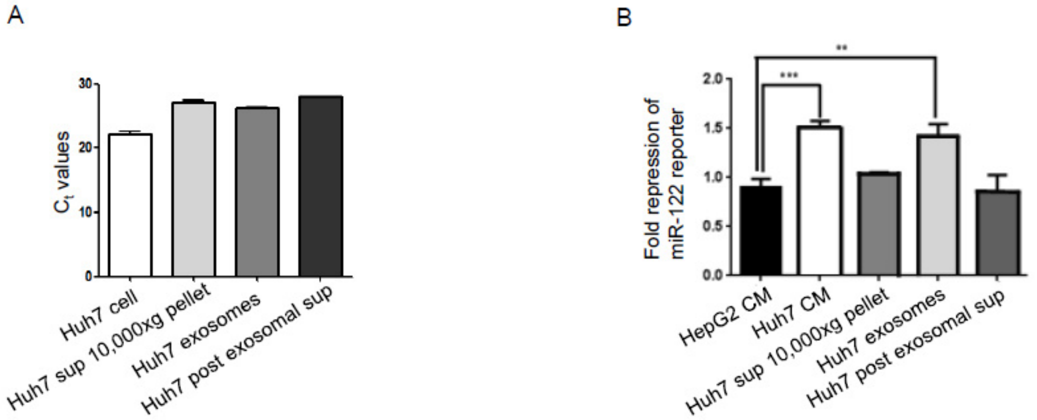

Figure S2 miR-122 present only in exosomes secreted by Huh7 cells are effectively delivered to HepG2 cells. **(A)** Huh7 Conditioned Media (CM) were centrifuged at 1,00,000g and separated into exosomal and post exosomal fractions. Out of 10ml of total Huh7 CM, RNA was extracted from 500 µl of post exosomal fraction. RNA was also extracted from the pellet which precipitated at 10,000g. These were then analyzed for miR-122 level by qPCR. We plotted the C_t values for each fraction and observed comparable levels of miR-122 in the equivalent amount of post exosomal and exosomal fractions. For this experiment n=3 and qPCR was carried out in triplicate. **(B)** Activity assays were then performed by adding cell equivalent amounts of Huh7 exosomes and post exosomal supernatant to reporter transfected HepG2 cells. Also the 10,000g pellet obtained during exosome isolation was also dissolved in medium and added to the reporter transfected HepG2 cells. We observed that miR-122 mediated repression increases in HepG2 cells only when Huh7 exosomes are added. n=3 and P values were calculated by unpaired t test

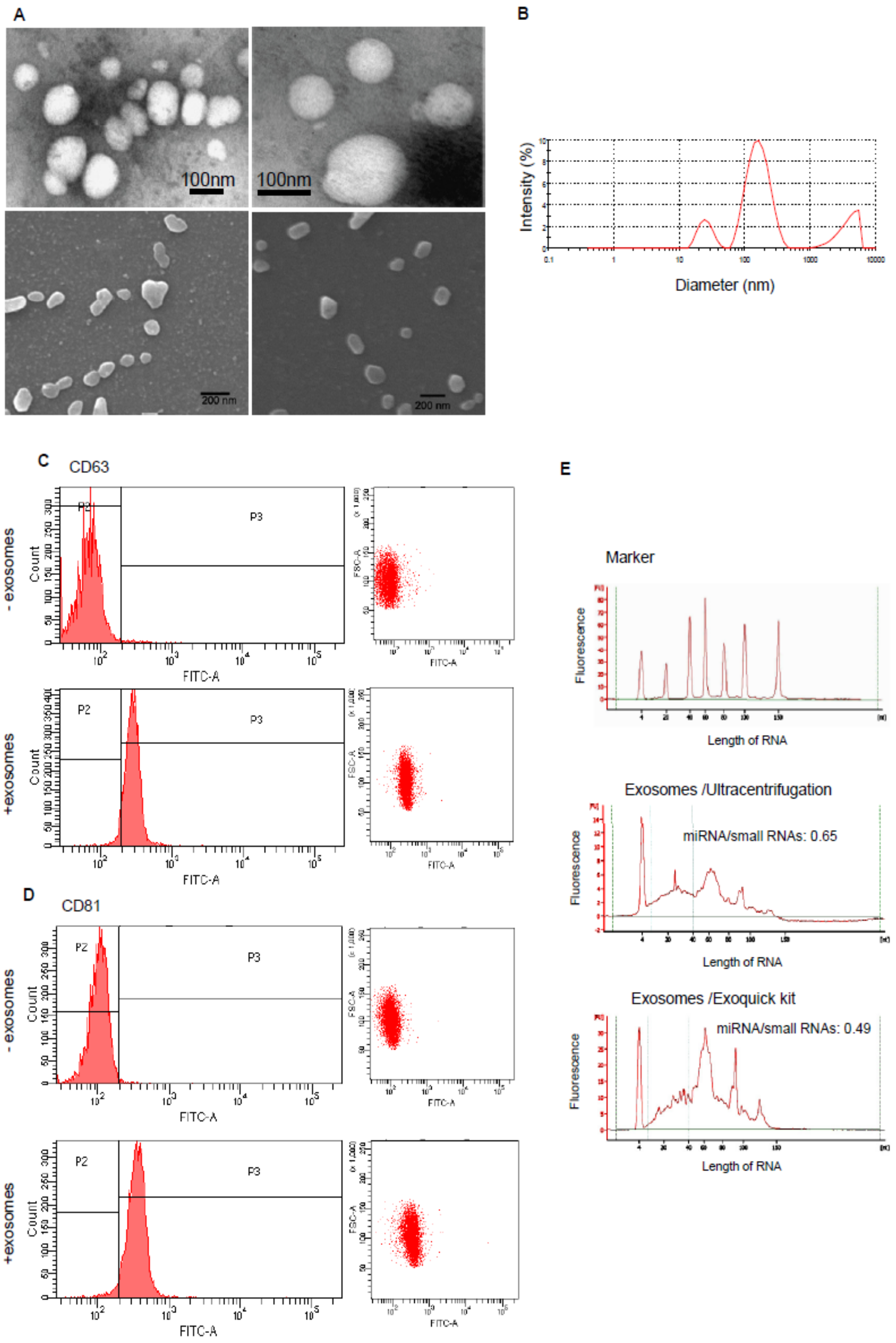

Figure S3 Biophysical and biochemical characterization of Huh7 exosomes. (A) Transmission and Scanning Electron micrograph of purified Huh7 exosomes isolated by ultracentrifugation at 1,00,000g. Exosomes were visualized by a Transmission electron microscope without any stain (*upper panels*). Scanning electron micrographs of exosomes visualized after gold coating (*lower panels*). (**B**) Dynamic Light Scattering analysis of Huh7 exosomes obtained as in (A). (**C-D**) Biotinylated antibodies against the tetraspanins CD63 or CD81 were immobilized on magnetic streptavidin beads. These were then used to pull down respective protein containing vesicles from exoquick precipitated Huh7 exosomes. Addition of Exo-FITC universal exosomes stain enabled the exosomes to be visualized by FACS. In the presence of added Huh7 exosomes there is a shift in FITC fluorescence indicating presence of the biochemical markers on the exosomes. **(E)** Exosomal small RNA analysis. Exosomes were isolated by the exoquick method and by the ultracentrifugation method at 100,000g. Exosomal RNA was then isolated from the two samples and compared for small RNA profile in an Agilent 2100 Lab-on–a-Chip Bioanalyzer (Agilent Technologies), using the Agilent Small RNA chip and reagent kit. The instrument software quantitated the peak area between 0 and 285 nt as small RNA region, the area within 10 to 40 nt as microRNA region, and provided percentages of miRNA detected for each sample. Exosomes isolated by ultracentrifugation showed a higher miRNA / small RNA ratio (65%) than those isolated by the exoquick method (49%). M indicates the RNA marker.

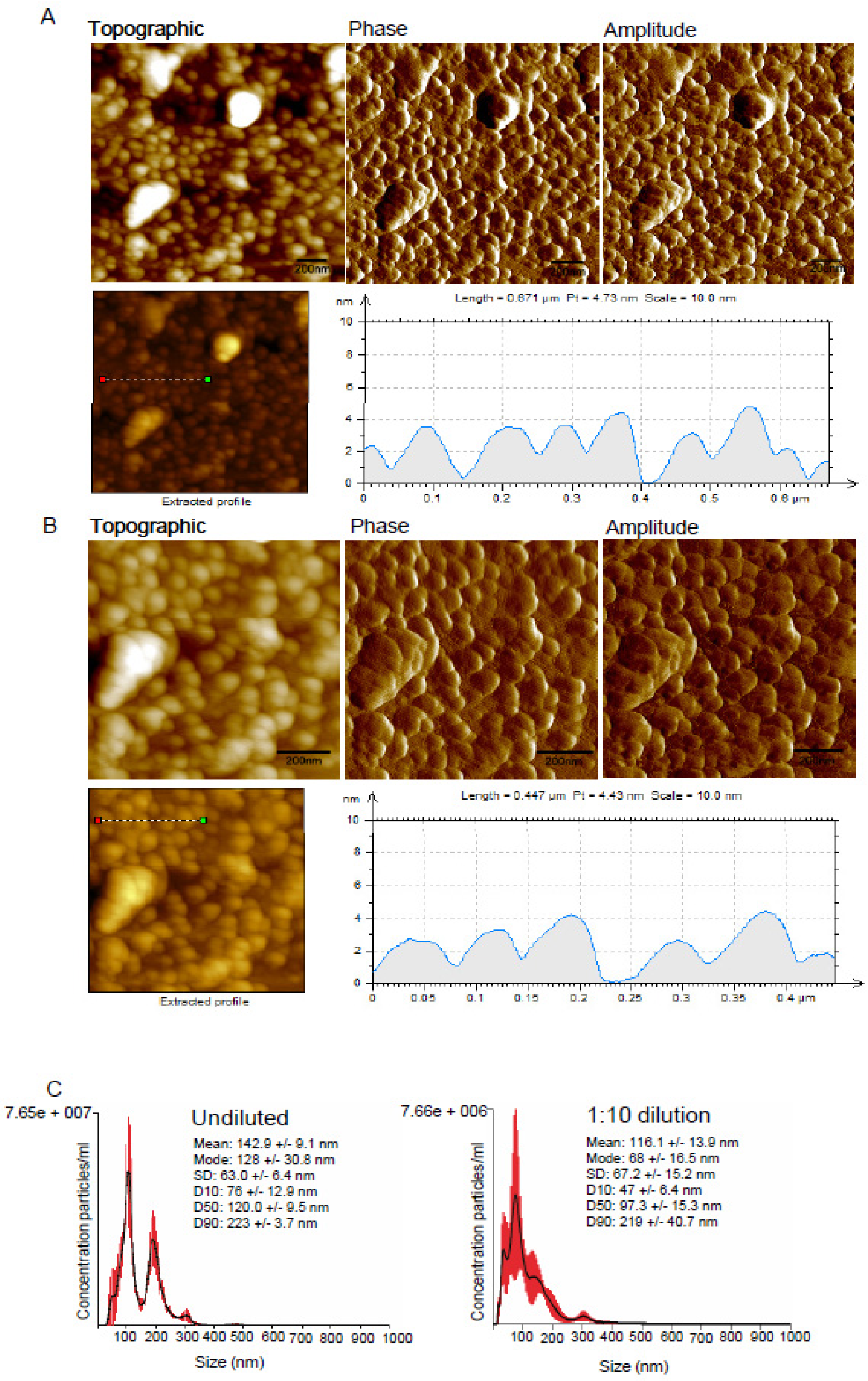

Figure S4 Size determination of Huh7 exosomes isolated by ultracentrifugation

(A) Tapping mode topographic, phase and amplitude AFM images showing round morphology of isolated exosomes. Lower panel shows the cross section profile of the dotted area indicating diameter of individual particles. **(B)** Zoomed in AFM images of the field shown in (A). Lower panel shows the cross section profile of the dotted area indicating diameter of individual particles. **(C)** Nanoparticle tracking analysis (NTA) of exosomes secreted by Huh7 cells isolated by ultracentrifugation at 1,00,000xg. Size and particle distribution plots of isolated exosomes before and after 10 fold dilution. Data are presented as the average and standard deviation of the three video recordings. The FTLA size distribution of the vesicles at various dilutions are mentioned. Lower values upon dilution seem to be consistent with decreased aggregates of individual vesicles at diluted concentrations.

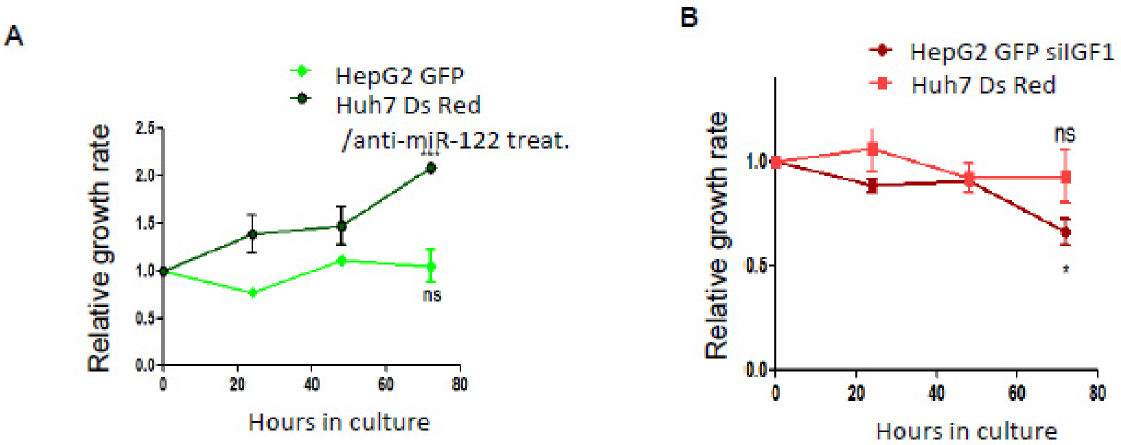

Figure S5 Effect of miR-122 depletion in Huh7 and IGF1 depletion in HepG2 cells on growth of neighboring cells. (A) Ds Red-Huh7 cells transfected with anti-miR-122 oligonucleotides and GFP- HepG2 cells were cocultured with each other for various time points. As a control the cells were also cultured separately for the same time durations and then mixed together. Cells harvested at each time point were fixed and analysed by FACS to detect the percentage of DsRed and GFP cells. The relative growth rate was then plotted by dividing the percentage of GFP or DsRed positive cells in the cocultured samples with that of the control samples. The downward trend of HepG2 growth upon coculture with Huh7 is disrupted at the 48 hour time point with a growth rate closer to 1. However at 72 hours of coculture the growth rate decreases slightly, probably due to the loss of the transfected anti-miR-122. P values have been calculated between the 0 and 72 h time points by paired t test. For each experiment, n=3. **(B)** GFP-HepG2 cells transfected with siRNAs against IGF1 were co-cultured with DsRed Huh7 cells for various time points. As a control the cells were also cultured separately for the same time durations and then mixed together. Cells harvested at each time point were fixed and analysed by FACS to detect the percentage of DsRed and GFP cells. The relative growth rate was then plotted by dividing the percentage of GFP / Ds Red positive cells in the cocultured samples with that of the control samples. We found that the increased growth rate of co-cultured DsRed Huh7 cells observed in Figure 2A was disrupted when co-cultured with IGF1 depleted HepG2 cells. P values have been calculated between the 0 and 72 h time points by paired t test. For each experiment, n=3.

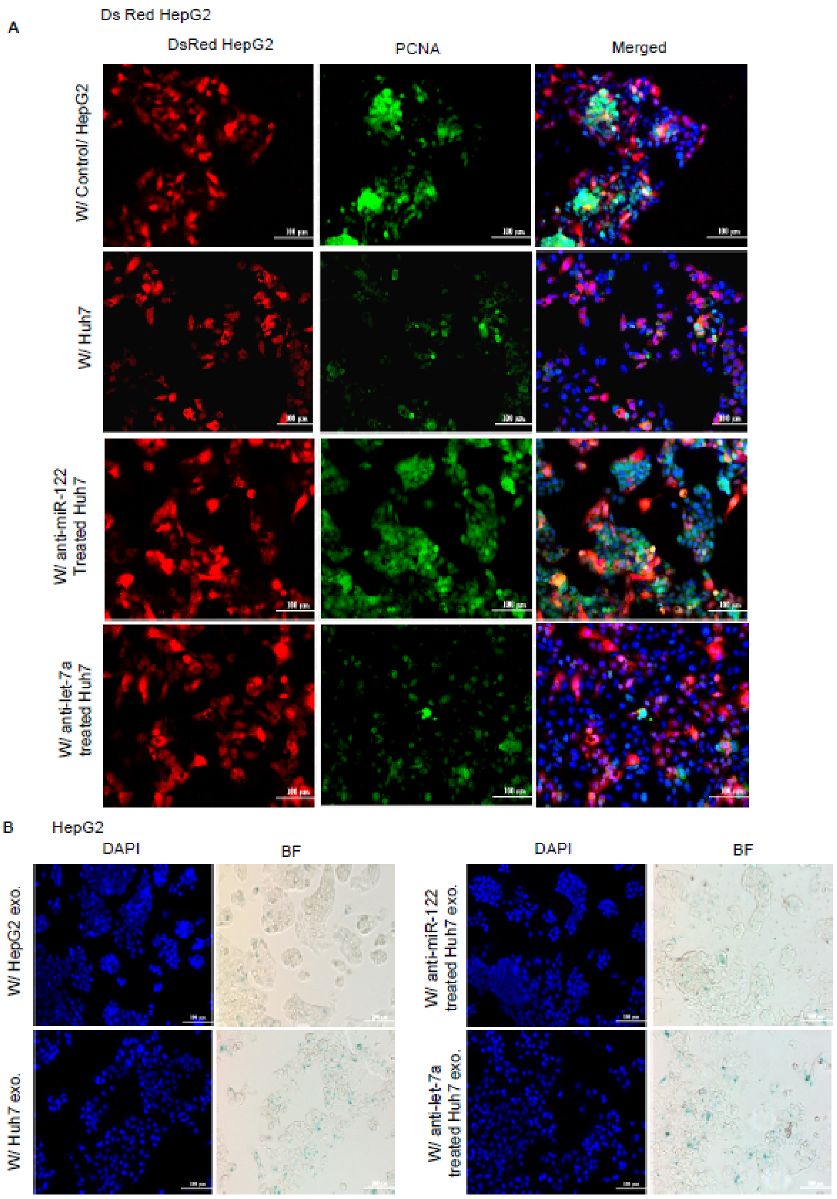

Figure S6 HepG2 cells show decreased proliferation and increased senescence in co-culture with Huh7 (A) PCNA staining of HepG2 cells stably expressing DsRed cocultured with either HepG2 (control), Huh7, anti-miR-122 oligonucleotide transfected Huh7 or anti-let7a transfected Huh7. Indirect immunofluorescence was done for PCNA antigen. **(B)** Senescence detection in HepG2 cells incubated with exosomes from HepG2 (control), Huh7, anti-miR-122 transfected Huh7 or anti-let7a transfected Huh7 cells. The number of senescent cells was documented by β-galactosidase staining following the protocol provided with the kit from Sigma.

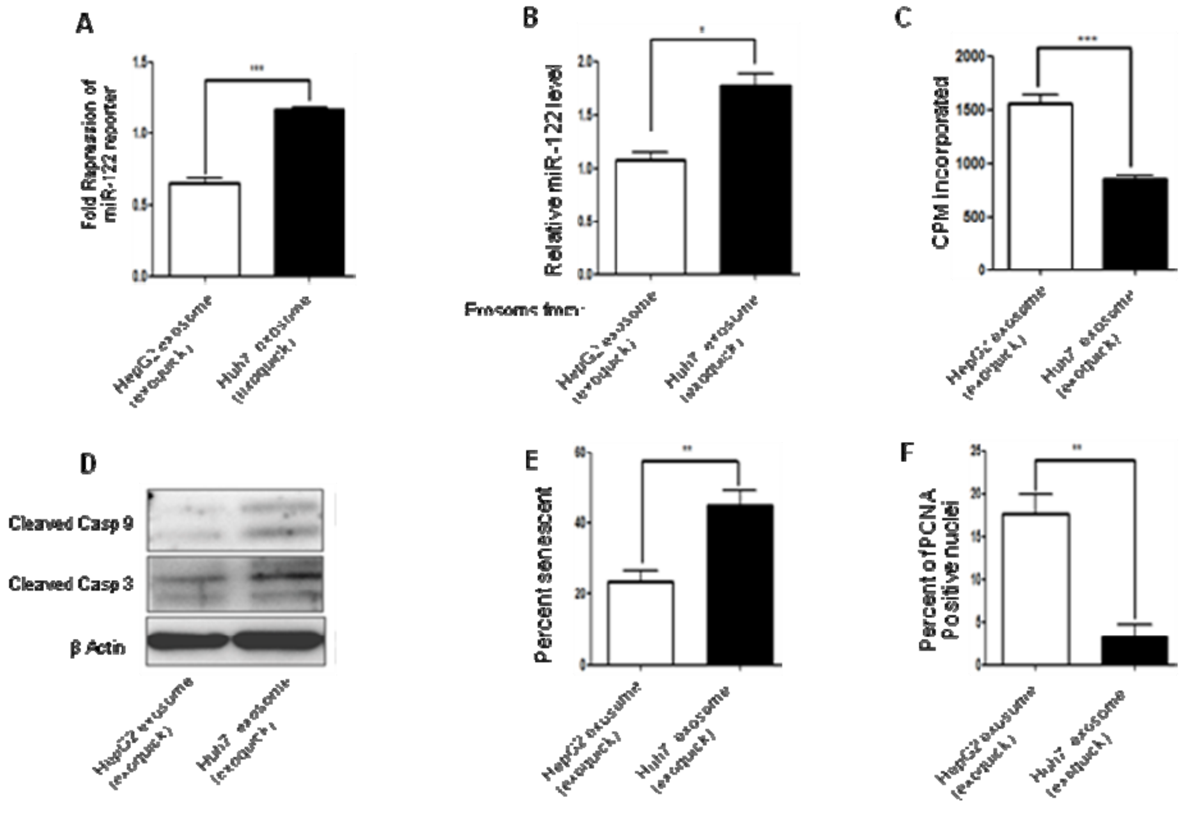

Figure S7 Effect of affinity purified Huh7 exosomes on HepG2

Huh7 and HepG2 exosomes were affinity purified using biotinylated antibodies based on the presence of their biochemical markers (CD 63, CD9, CD81) by using the Exoflow kits (described in methods section of the text) and a combination of antibodies against CD9, CD 63 and CD81. The eluted exosomes were then added to HepG2 cells to redo the experiments completed with exosomes isolated by differential ultracentrifugation. **(A)** Fold repression of miR-122 reporter in HepG2 cells incubated with exosomes from HepG2 and Huh7 cells. Experiment was done in triplicate and P value was calculated by unpaired t test. **(B)** Relative miR-122 level of HepG2 cells used in experiments described in panel A. N=3 and P value was calculated by Paired t test. **(C)** [^{3}H] Thymidine incorporation of HepG2 cells incubated with exosomes from Huh7 and HepG2 cells. N=4, P value was calculated by unpaired t test. **(D)** Immunoblots showing Cleaved PARP and Cleaved Caspase 3 and 9 in HepG2 cells incubated with Huh7 exosomes and 50µg/ml of doxorubicin. Incubation with Huh7 exosomes was done for 48 hours and doxorubicin was added for the last 24 hours. **(E)** HepG2 cells were incubated with HepG2 and Huh7 exosomes and the percentage of senescent cells were detected. The number of senescent cells was documented by β-galactosidase staining following the protocol provided with the kit from Sigma. Data represent 4 fields from 2 independent experiments. **(F)** HepG2 cells were incubated with Huh7 exosomes and the percentage of PCNA positive nuclei were identified. Data represent 4 fields from 2 independent experiments.

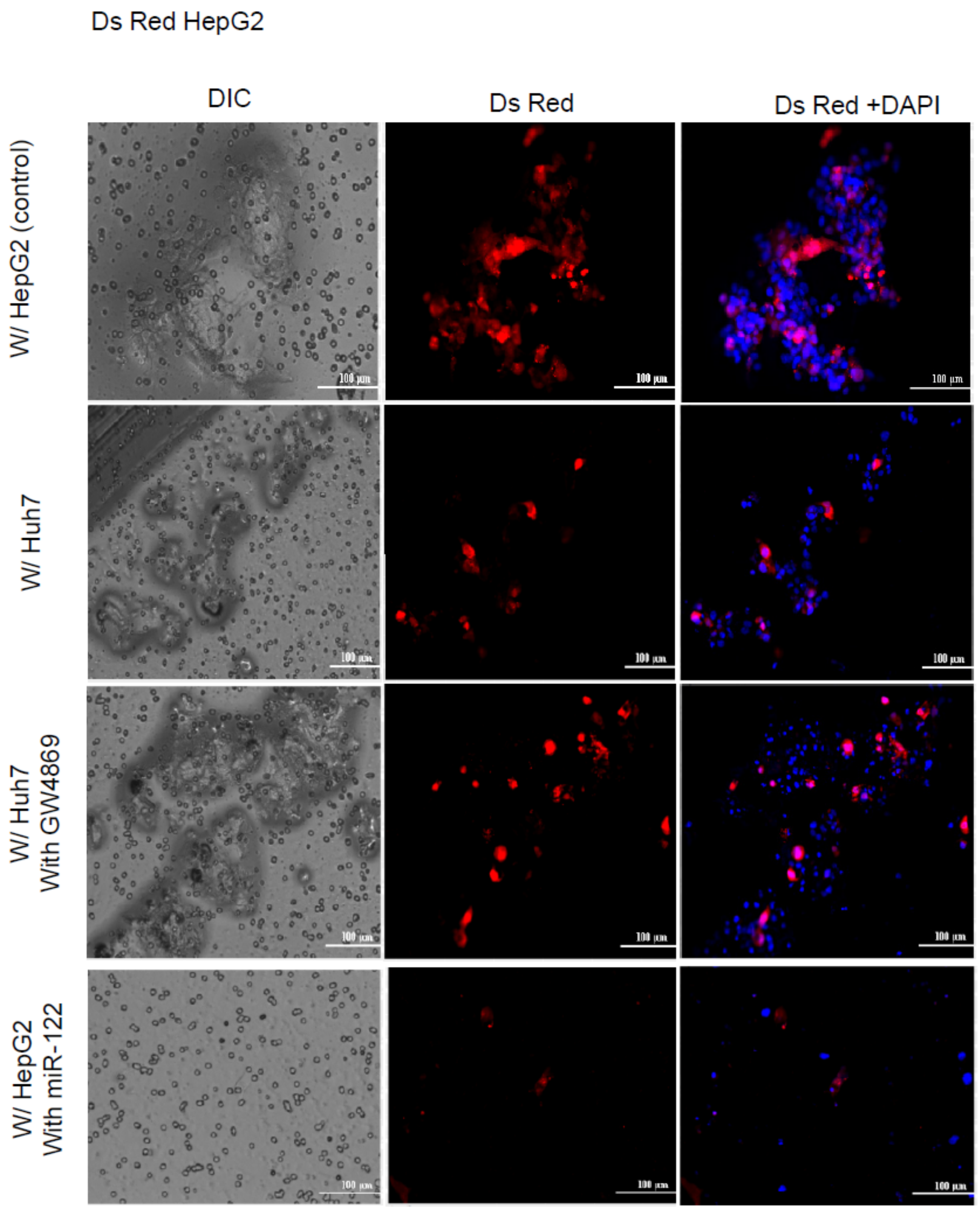

Figure S8 HepG2 cells show reduced invasion through matrigel in presence of Huh7.

Microscopic images of DsRed positive HepG2 cells invaded through matrigel when co-cultured with either HepG2 cells (control), Huh7 cells (in the presence and absence of the exosome inhibitor GW4869) and HepG2 cells expressing miR-122. The cells were grown on polycarbonate membranes having a pore size of 8µm on a layer of matrigel. After 48h, the number of DsRed positive cells invaded to the other side of the membrane was documented. DAPI stained the nucleus of all invaded cells.

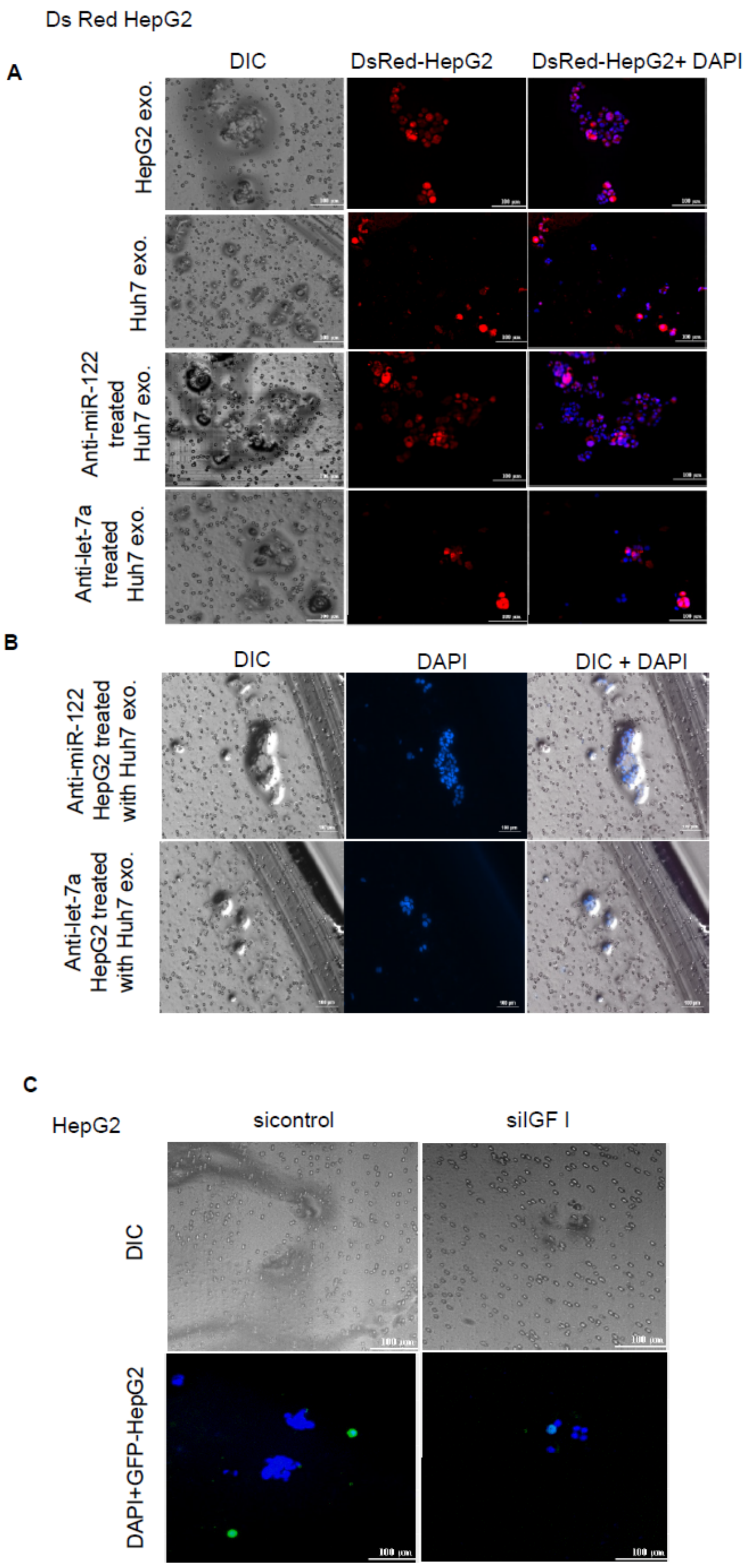

Supplementary Figure S9 HepG2 cells show reduced invasion through matrigel in presence of miR-122 containing exosomes

(A-B) Microscopic images of DsRed positive HepG2 cells invaded through matrigel after incubation with exosomes from HepG2 (control), Huh7 and anti-miR-122 or anti-let-7a transfected Huh7 cells (A). In panel B, HepG2 cells which were transfected with anti-miR-122 and anti-let7 oligonucleotides and then incubated with Huh7 exosomes. The cells were grown on polycarbonate membranes having a pore size of 8 µm on a layer of matrigel. 24 h after treatment with the exosomes, the number of DAPI positive DsRed cells on the other side of the membrane was documented. **(C)** Images of invaded GFP positive HepG2 cells which were transfected with either siIGF1 or siControl and seeded on a layer of Huh7 cells on matrigel. The number of HepG2 cells invading through the layer of Huh7 and matrigel was then visualized by staining the other side of the membrane with DAPI.

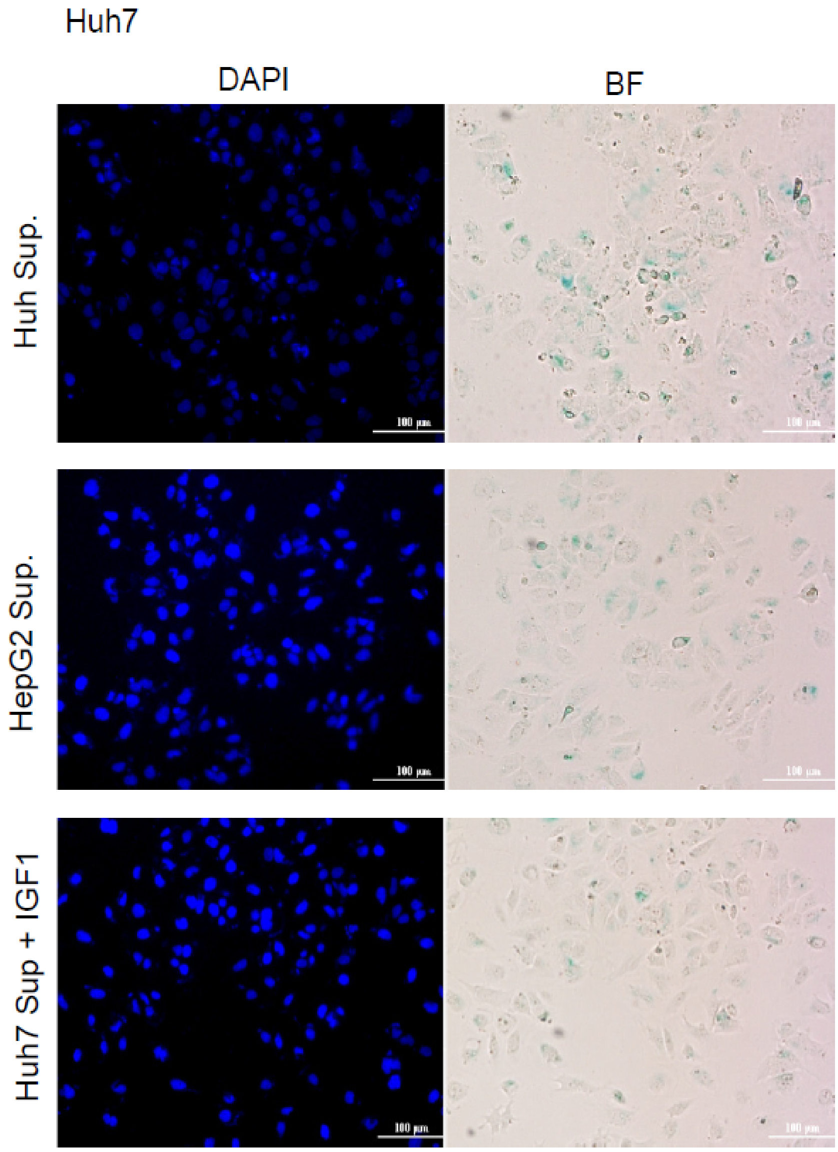

Figure S10 Huh7 cells show reduced senescence in the presence of IGF1

Microscopic images of senescent Huh7 cells after incubation with CM from HepG2 cells or with exosome depleted CM from Huh7 cells containing 0 and 100ng/ml of IGF1 for 48 h. The senescent cells were visualized by β-galactosidase staining.

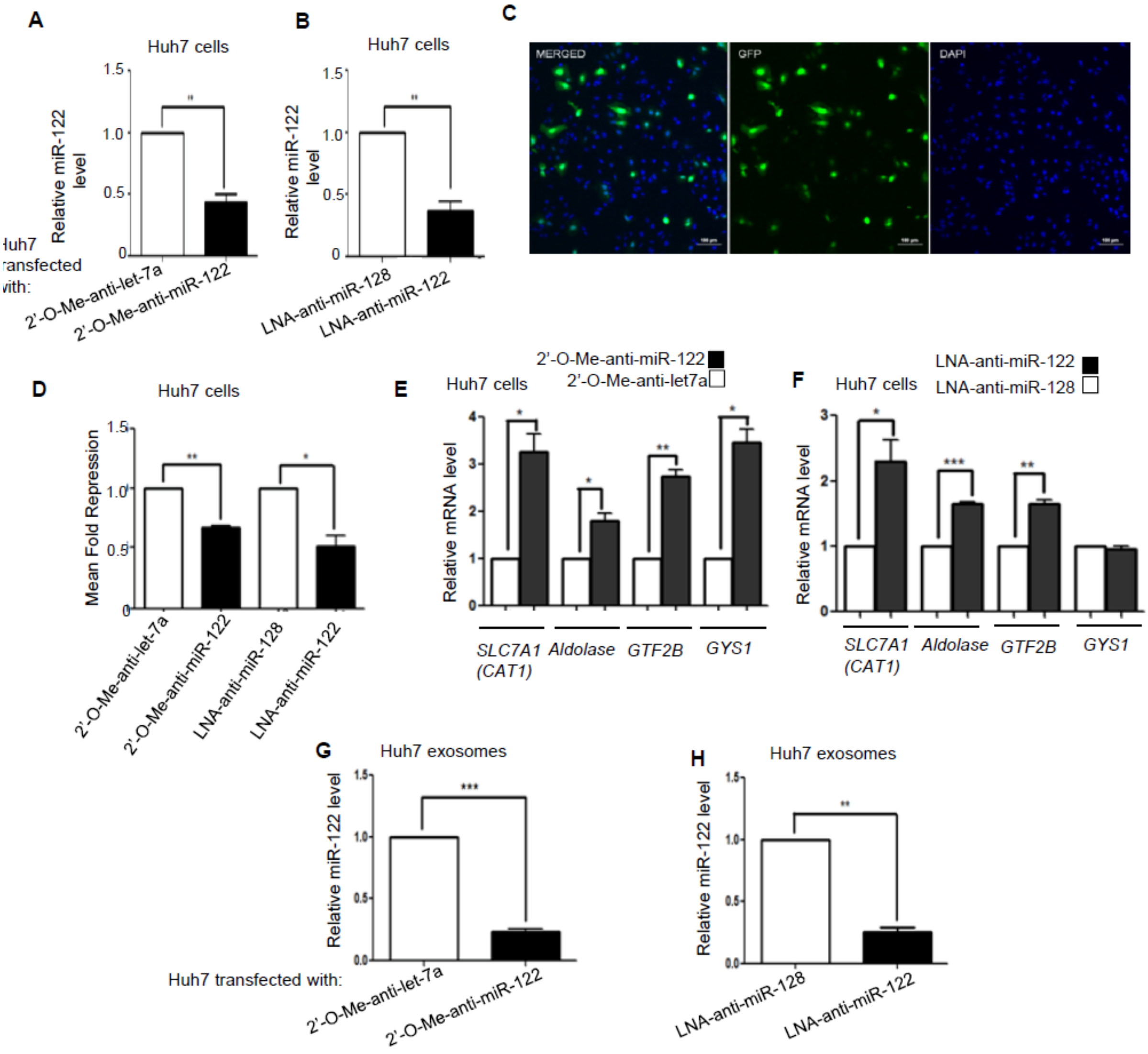

Figure S11: Transfection of Huh7 cells with anti-miR-122 oligonucleotides decreases cellular and exosomal miR-122 levels

(A and B) Huh7 cells transfected with 2'-O-Methyl (A) or LNA^{TM}modified (B) -anti-miR-122 oligonucleotides showed reduced levels of cellular miR-122 compared to anti let-7a and anti-miR-128 transfected cells (controls). qPCR was performed in triplicate from a minimum of three biological replicates and data are represented to show relative level of cellular miRNA level. Statistical significance was calculated by paired t test. **(C)** Low transfection of Huh7 cells. Huh7 cells were transfected with a pCIneo plasmid encoding GFP and after 48h of transfection number of green cells were calculated to get the transfection efficiency. DAPI stained nucleus are shown in blue. The average transfection efficiency of Huh7 cells came to 20%. This was calculated on the basis on three individual transfection events. **(D)**

Luciferase assays with Huh7 cells expressing miR-122 reporter showed reduced miR-122 mediated repression in 2'-O-Methyl anti-miR-122 and LNA^{TM}-anti-miR-122 transfected cells with respect to controls. **(E) and (F)** RT-qPCR based quantification revealed higher levels of miR-122 targets in anti-miR-122 oligonucleotide transfected Huh7 cells with respect to controls. Data shown above are indicative of three biological replicates and qPCR for each was performed in triplicate. P values were calculated by paired t test. **(G-H)** Huh7 cells transfected with anti-miR-122 oligonucleotides showed reduced levels of exosomal miR-122 in exosomes isolated from the supernatant of the corresponding cells. Huh7 cells were transfected with anti-miR-122 or control oligos. Exosomes were isolated from these cells 48 hours after media change following transfection and RT-qPCR assays revealed reduced levels of exosomal miR-122 in anti-miR-122 transfected cells. Data shown above are inclusive of four biological replicates and qPCR for each was performed in triplicate. P values were calculated by paired t test.

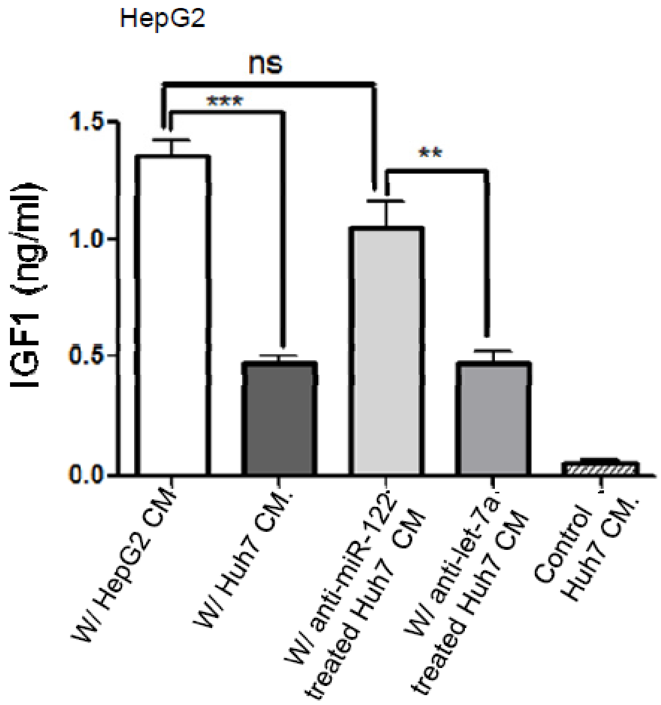

Figure S12 Huh7 released exosomes decreases IGF1 production of HepG2 cells

Levels of IGF1 secreted by HepG2 cells when incubated with Huh7 CM. HepG2 cells were incubated for 48 h with CM from Huh7 and Huh7 transfected with anti-miR-122 and anti-let-7a (control) oligonucleotides. IGF1 levels in cell free supernatants were measured by quantitative ELISA using 'Quantikine Immunoassay for Human IGF1' (R&D System Catalog no. DG100). Huh7 CM kept at 37°C for 48 hours without cells was used as negative control and HepG2 CM was used as a positive control. Data was normalized with IGF1 present in FCS containing media used to culture the cells. Graph represents 4 experiments and p values were calculated using unpaired t test.

Supplementary methods

Immunofluorescence

For immunofluorescence, cells were grown on gelatin coated coverslips and were fixed with 4% paraformaldehyde for 30 mins, washed with 1 X PBS twice to remove the paraformaldehyde. Cells were then blocked and permeabilized using 1XPBS containing 10% goat serum, 1% Bovine Serum Albumin (BSA), and 0.1% Tritin-X-100 for 30 mins at room temperature. Primary antibody incubation was done in 1XPBS with 1% BSA at 4ºC overnight in a humid chamber. The anti-mitosin antibody was used at a dilution of 1:100. Secondary antibody incubation was done in 1XPBS with 1% BSA for 1h at room temperature. Secondary anti-rabbit antibodies labeled either with Alexa Fluor® 488 dye (green) or Alexa Fluor® 594 dye (red) fluorochromes (Molecular Probes) were used at 1:500 dilutions. Coverslips were then mounted with Vectashield containing DAPI and observed under a fluorescence microscope. Images were captured with a Nikon Eclipse Ti microscope. All post capture analysis and processing were done using Nikon NIS-Element AR 3.1 software.

Western blotting

Cells were lysed in 1X Passive Lysis Buffer (PLB) (Promega) and quantified using Bradford reagent (Thermo Scientific). Samples were assayed as per standard protocol . Imaging of all western blots was performed using an UVP BioImager 600 system equipped with VisionWorks Life Science software (UVP) V6.80. Details of all antibodies used are given at the bottom.

Cell senescence assay

Cells were assayed for senescence using Senescence Cells Histochemical Staining Kit from Sigma as per manufacturers' protocol. Assayed cells were thoroughly washed with PBS and finally mounted on a slide with Vectashield containing DAPI (H-1200, Vector Laboratories) for

observation with a motorized Nikon Eclipse Ti microscope equipped with 10X Plan Fluor 10X/0.30 objective. Images were captured using a Nikon Ri1 camera.

Invasion assays

Using sterile forceps 24 well cell culture inserts from Nunc (Catalog no. 140629) were removed from their packaging and put in to wells of a 24- well plate. Pre-cooled inserts were washed twice with cold DMEM and then 100µl of 1:6 diluted Matrigel (BD catalog no. 354248) was coated onto the centre of each insert using pre-cooled pipette tips. The matrigel was spread across the entire surface and the coated inserts were kept at 37°C to solidify. For Figure 6C, matrigel with reduced amounts of growth factors were used. (BD catalog no. 354230).

For cell seeding, 24h after transfection cells were collected by trypsinization and $4X10^4$ of transfected HepG2 cells, along with the cell used for co-culture, were seeded on to the upper chamber of the cell culture insert in 200µl of culture medium. 300µl of medium was placed in the lower chamber and cells were cultured for 48 h. For Figure 6C, $1X10^5$ of nontransfected Huh7 cells were seeded to form a layer on the reduced growth factor matrigel coated cell culture insert. $4x10^4$ GFP transfected HepG2 cells were then seeded on top of that. After 24h, medium was aspirated from the lower chamber and replaced by 500µl of 4% Paraformaldehyde. This was kept for 30 mins. After 30 mins, the medium from the inner chamber was aspirated out and and the matrigel layer was removed by wiping with a moist cotton swab. The insert was taken and the membrane was cut out using a sharp blade. The membrane was mounted on DAPI such that cells on the outer surface were stained with DAPI. The number of DAPI and GFP/DsRed positive cells was counted under the fluorescence microscope. The percentage of GFP/DsRed cells from the total DAPI positive invaded cells were then determined.

Cell proliferation assay

$5x10^4$ cells were seeded in each well in a 24 well plate and incubated in a 37°C humidified 5% CO_2 incubator for 24 h. Cells were then washed with serum free media (DMEM) and then 1 ml of DMEM was added to each well to synchronize cells in a low growth state. After 24h media was replaced with serum containing media with the added exosomes. 24h afterwards 1µCi of ^{3}H thymidine was added to each well. Cells were incubated for 8 h in a 37°C humidified 5% CO_2 incubator.

To extract ^{3}H-thymidine labelled DNA, cells were washed with 1 ml of ice-cold PBS. Then 1 ml of ice-cold 5% TCA was added and cells were incubated at 4°C for 30 mins. After 30 mins, TCA solution was aspirated out and cells were washed once with PBS. 0.5 ml of 0.5M NaOH/0.5% SDS solution was added at room temperature to the cells. The cell suspension was pipetted up and down to lyse the cells. The mixture was then added directly to scintillation vials containing scintillation fluid. The vials were counted in a scintillation counter for assessment of radionucleotide incorporation.

Animal handling and mouse primary hepatocyte isolation

Animals were obtained from the animal house of the institute and all experiments were performed according to the guidelines set by Institutional Animal Ethics committee following the Govt. of India regulations. Mouse primary hepatocytes were isolated using the hepatocyte product line from Gibco Invitrogen Corporation. Adult Balb/c mice (4-6 weeks) were anaesthetized and the portal vein was cannulated using a 25G butterfly cannula and an incision was made in the inferior vena cava. The liver was perfused with 350 mL of warm (37°C) Liver Perfusion Medium (Cat. No. 17701) at a rate of 35 mL/minute with the perfusate exiting through the severed vena cava. This was followed by a Collagenase-Dispase digestion with Liver Digest Medium (Cat no. 17703) at a rate of 35 mL/minute. The liver was then aseptically transferred to the tissue culture hood on ice in Hepatocyte Wash Medium (cat no. 17704). Using blunt forceps the digested liver was torn open to release the hepatocytes. Cell clumps were dissociated by

gently pipetting the solution up and down using a 25ml pipette. The solution was then filtered through 100 µM nylon caps atop 50 ml conical tubes. The cell suspension was then centrifuged at 50 x g for 3 min. The pellet was gently resuspended in 10 ml of Wash Medium using 25 ml pipette and the centrifugation repeated.

Cells were finally resuspended in Hepatocyte Wash Medium with 10% FCS and plated at $1x10^7$ cells /ml. Cells were plated in tissue culture treated collagen (Gibco Cat. No. A10483-01) coated plates at 12.5µg/cm^2. Unattached cells are poured off 4 h after plating and medium was replaced with Hepatozyme-SFM (Cat no. 17705) with glutamine and 1% Pen/Strep. For Figure 5F, IGF1 was added at this stage and kept for 48 h.

Exosome isolation using Exoquick and FACS analysis of affinity purified exosomes

Huh7 supernatant taken from 1.2 x 10^7 cells (20ml of supernatant) was centrifuged at 2000g for 15 minutes to remove cellular debris. Exoquick™ (System Biosciences Catalog No. EXOQ20A-1) was added to the Conditioned Media (CM) as per manufacturers' protocol. This was kept overnight at 4ºC and exosomes were precipitated by centrifugation at 1500xg for 30 minutes at 4ºC. These were then resuspended in 500µl of PBS. Streptavidin magnetic beads were coated with biotinylated capture antibodies as per manufacturers' protocol (catalog no. EXOFLOW150A-1). 200µl of the Exoquick precipitated Huh7 exosomes were added to the beads and kept at 4ºC overnight for affinity interaction. Beads containing pulled down exosomes were then washed (as per manufacturers' protocol) and stained with the Exo-FITC universal exosome stain provided in the kit. Stained exosomes were then visualized on a BD LSR Fortessa FACS machine. Affinity purified exosomes were also eluted from the beads (as per manufacturers' protocol) and experiments were done with the eluted vesicles (Figure S6).

Transmission Electron microscopic imaging of Huh7 exosomes

Exosomes of Huh7 cells obtained after ultracentrifugation of cell culture supernatants (exosomes from ~6x10^6 cells resuspended in 1ml PBS) were resuspended in PBS and 10μl spotted onto Formvarcoated grids (200 mesh). The exosomes were then directly visualized. Grids were examined by a FEI Technai G^2 Spirit BioTWIN electron microscope at 100 kV.

Scanning electron microscopic imaging of Huh7 exosomes

Exosomes of Huh7 cells obtained after ultracentrifugation of cell culture supernatants were resuspended in PBS (exosomes from ~6x10^6 cells resuspended in 1ml PBS) and spotted onto sample slides. The sample slides were dried completely under Critical Point dryer (Quorum Technologies) after drying the samples were kept on the sample holder by using carbon tape. Gold coating was done with the current 10 mA at 10^{-6}-10^{-8} mbar/Pa (Quorum Technologies, Model No. SC7620). SEM imaging was performed using TESCAN Vega II LSU (TESCAN Digital Microscopy Imaging). Imaging and measurements were done by Vega TC software.

Atomic force microscopic imaging of Huh7 exosomes

5ul of Huh7 exosomes obtained from Huh7 cells (exosomes from ~6x10^6 cells resuspended in 1ml PBS) and isolated by ultracentrifugation were resuspended in PBS and deposited onto freshly cleaved muscovite Ruby mica sheet (ASTM V1 Grade Ruby Mica from MICAFAB, Chennai) for 15-30 minutes , Mica sheets are negatively charged so that the molecule binds strongly to the mica surface. The sample was dried by using vacuum dryer for 15 minutes. The sample slides were then gently washed with 0.5 ml MilliQ water to remove molecules that were not firmly attached to the mica and dried as mentioned above. AAC mode AFM was performed using a Pico plus 5500 ILM AFM (Agilent Technologies USA) with a piezoscanner maximum range of 9um. Micro fabricated silicon cantilevers of 225 μm in length with a nominal spring force constant of 21-98 N/m were used from Nano sensors, USA. Cantilever oscillation

frequency was tuned into resonance frequency. The cantilever resonance frequency was 150-300 kHz. The images (256 by 256 pixels) were captured with a scan size of between 0.5 and 5 um at the scan speed rate of 0.5lines/S. Images were processed by flatten using Pico view1.1 version software (Agilent Technologies, USA). Image manipulation has been done through Pico Image Advanced version software (Agilent Technologies, USA).

Nanoparticle Particle Tracking analysis

Huh7 exosomes (from ~4 x 10^6 cells) isolated by ultracentrifugation at 1,00,000g were resuspended in 1 ml of PBS and were used for NTA. 700µl of the sample was loaded into the sample chamber of an NS500 unit (Nanosight, Amesbury, UK) and three videos of 30 seconds were recorded for each sample. The camera shutter speed was 39.997 milliseconds 24.99 fps, with camera gains of 400 and capture magnification 20X. Data analysis was performed with NTA 2.3.5 software (Nanosight). Software settings for analysis were: Detection Threshold: 1; Blur: Auto; Minimum expected particle size: 50 nm. Data are presented as the average and standard deviation of the three video recordings of each sample.

Dynamic light scattering of Huh7 exosomes

Exosomes of Huh7 cells obtained after ultracentrifugation of cell culture supernatants (~6x10^6 cells) were resuspended in 100µl PBS. Dynamic Light Scattering was then done in a Malvern Zetasizer Nano (model Zen 3600).

ChIP Assay

ChIP assay was done as described previously (3). Shortly, 1% formaldehyde was added directly to the medium for 15 min to cross-link nuclear proteins to DNA. Glycine was added to final concentration 0.125 M and incubated for 5 min to avoid over-crosslinking. After PBS wash, cells were collected in ice-cold PBS supplemented with a protease inhibitor cocktail. Cells were lysed

with lysis buffer (1% SDS, 10 mM EDTA, protease inhibitors and 50 mM Tris–HCl, pH 8.1) and the lysates were sonicated to obtain DNA fragments of 300–1000 bp in length. Cellular debris was removed by centrifugation and the lysates were diluted 1:10 in ChIP dilution buffer (0.01% SDS, 1.1% Triton X-100, 1.2 mM EDTA, 167 mM NaCl, protease inhibitors and 16.7 mM Tris–HCl, pH 8.1). Non-specific background was removed by incubating the chromatin suspension with a salmon sperm DNA/protein G agarose (Invitrogen) for 30 min at 4°C with agitation. The samples were centrifuged and the recovered chromatin solutions were incubated with indicated antibodies overnight at 4°C with rotation. Antibody details have been given in the 'List of Antibodies used'. The immuno-complexes were collected with 60 µl of protein G agarose (Invitrogen) for 2 h at 4°C with rotation. The beads were washed sequentially for 5 min by rotation with 1 ml of the following buffers: low-salt wash buffer (0.1% SDS, 1% Triton X-100, 2 mM EDTA, 150 mM NaCl and 20 mM Tris–HCl, pH 8.1), high-salt wash buffer (0.1% SDS, 1% Triton X-100, 2 mM EDTA, 500 mM NaCl and 20 mM Tris–HCl, pH 8.1) and LiCl wash buffer (0.25 mM LiCl, 1% Triton X-100, 1% sodium deoxycholate, 1 mM EDTA and 10 mM Tris–HCl, pH 8.1). Finally, the beads were washed twice with 1 ml TE buffer (1 mM EDTA and 10 mM Tris–HCl, pH 8.0). The cross-linking was reversed by adding Chelex-100 and incubated for 10 min at 100°C. The remaining proteins were digested by adding proteinase K (final concentration 40 µg/ml) and incubated for 30 min at 55°C. The DNA was recovered by phenol/chloroform/isoamyl alcohol (25:24:1) extractions and precipitated with 0.1 vol of 3 M sodium acetate, pH 5.2 and 2 vol of ethanol using glycogen as a carrier.

Post Capture Image analysis

All western blot were processed with Adobe Photoshop CS4 for all linear adjustments and cropping. All images captured on Nikon Eclipse Ti microscope were analyzed and processed with Nikon NIS ELEMENT AR 3.1 software. Cropping was done using Adobe Photoshop CS4.

Supplementary References

1. Pillai, R.S., Bhattacharyya, S.N., Artus, C.G., Zoller, T., Cougot, N., Basyuk, E., Bertrand, E. and Filipowicz, W. (2005) Inhibition of translational initiation by Let-7 MicroRNA in human cells. *Science*, 309, 1573-1576.
2. Ghosh, J., Bose, M., Roy, S. and Bhattacharyya, S.N. (2013) Leishmania donovani targets Dicer1 to downregulate miR-122, lower serum cholesterol, and facilitate murine liver infection. *Cell host & microbe*, 13, 277-288.
3. Xu, H., He, J.H., Xiao, Z.D., Zhang, Q.Q., Chen, Y.Q., Zhou, H. and Qu, L.H. (2010) Liver-enriched transcription factors regulate microRNA-122 that targets CUTL1 during liver development. *Hepatology*, 52, 1431-1442.
